# Coexistence of Microplastics and Heavy Metals in Lake Sediments: Interaction Mechanisms and Complex Ecological Risks

**DOI:** 10.3390/toxics14080731

**Published:** 2026-08-18

**Authors:** Jiahui Mi, Junping Lu, Zhuo Li, Yongqin Jia

**Affiliations:** 1 Water Conservancy and Civil Engineering College, Inner Mongolia Agricultural University, Hohhot 010018, China; yangzhiyuzhong@163.com (J.M.); lxz60138@163.com (Z.L.); 2 State Key Laboratory of Water Engineering Ecology and Environment in Arid Area, Inner Mongolia Agricultural University, Hohhot 010018, China; 3 State Gauge and Research Station of Wetland Ecosystem, Wuliangsuhai Lake, Bayan Nur 014404, China; 4 Autonomous Region Collaborative Innovation Center for Integrated Management of Water Resources and Water Environment in the Inner Mongolia Reaches of the Yellow River, Hohhot 010018, China

**Keywords:** lake sediment, microplastics, heavy metals, response mechanism, combined pollution

## Abstract

Lake sediments act as important sinks for heavy metals and microplastics, yet the mechanisms governing their enrichment, coexistence, and combined ecological risks remain insufficiently understood. This study investigated sediments from Daihai Lake, China, systematically characterizing the occurrence features of microplastics (morphology, size, and composition) and their associated heavy metal contents. A composite pollution risk framework (Multi Feature Potential Ecological Risk Index) was developed to evaluate microplastics–heavy metals interactions using correlation analysis, principal component analysis, and cluster analysis. In addition, a two-dimensional pollution index was applied to assess combined ecological risks. Results showed that MP abundance ranged from 6.60 to 26.80 n·g^−1^, with a decreasing trend from southwest to northeast. Microplastics were dominated by fragments, with a high proportion of small particles (<0.25 mm), and were mainly composed of polyethylene terephthalate and polypropylene. The average concentrations of heavy metals in sediments were ranked as follows: Mn (863 ± 78 mg·kg^−1^); Cr (124 ± 28 mg·kg^−1^); Zn (86 ± 17 mg·kg^−1^); Ni (43 ± 10 mg·kg^−1^); Cu (36 ± 0.1 mg·kg^−1^); Pb (23 ± 5 mg·kg^−1^); As (15 ± 4 mg·kg^−1^); and Cd (0.20 ± 0.04 mg·kg^−1^). The two-dimensional comprehensive index values ranged from 124 to 1032, with an average value of 365.0, exceeding the risk threshold (>100). Approximately 70% of sampling sites exhibited high composite pollution risks. Small-sized and fibrous microplastics showed significant positive correlations with multiple heavy metals, indicating strong carrier effects.

## 1. Introduction

Microplastics (MPs), as a new type of non-degradable environmental pollutant with a particle size of less than 5 mm along the longest axis, are ubiquitous in the environment and produce a series of ecological and environmental toxicity effects, making them a global environmental hotspot [1,2,3]. Researchers have found their traces in rivers, lakes, reservoirs, and oceans [4,5,6]. Most MPs undergo various complex reactions in natural environments, such as UV oxidation, mechanical abrasion, and biological degradation [7,8,9,10,11,12], which further reduce their size and make their environmental behavior more active [13]. Moreover, they can also cause changes in the surface texture and functional groups of MPs, significantly enhancing their adsorption capacity for coexisting pollutants such as heavy metals (HMs) and organic toxins [14]. MPs are continuously transferred to sediments through natural sedimentation, bioaccumulation, and particle aggregation, leading to their persistent accumulation [15,16]. Consequently, sediments serve as an important sink for MPs and a key medium in the migration, transformation, and ecological risk assessment of associated pollutants.

At the same time, HMs in lake environments, as persistent pollutants with “three causative” effects, will adhere to the surface of MPs [17]. The presence of MPs can not only serve as carriers of HMs, but also change the chemical behavior of HMs adsorbed on the surface through their unique physicochemical properties, inducing the mutual transformation between different forms of occurrence and producing ecological effects that are different from those of single pollutants [18]. As HMs pollution to the environment depends not only on the total amount, but also on its form, the environmental behavior and biological effects exhibited by HMs in different chemical forms will vary greatly [19]. Previous studies have shown that the unique surface characteristics of MPs can affect sediment deposition [17,20]. The interactions between MPs and HMs [21,22] contribute to the accumulation of pollutants in sediments, making the latter important sinks for pollutants and potential endogenous sources of pollutant release to overlying waters [23]. Some forms of HMs are directly absorbed by benthic plants and organisms, causing continuous accumulation in organisms [24]. When they enter the food chain, they pose a serious threat to other organisms and the ecological environment [20].

Lake sediments are important sinks of persistent pollutants, and the coexistence, interaction, and synergistic migration and transformation behavior of MPs and HMs in such media have become core topics in the study of composite pollution. Therefore, how to systematically characterize the co-distribution pattern of the two in sedimentary environments and accurately evaluate their combined pollution ecological risk effects is of great significance for revealing long-term pollution accumulation trends, predicting ecological risk evolution, and guiding regional environmental governance. At present, research on MPs and HMs pollution in lake sediments globally mainly focuses on the occurrence characteristics and distribution patterns of single pollutants, while research on the synergistic behavior and composite pollution risks of MPs and HMs is relatively insufficient. Common single pollutant indicator methods for ecological risk assessment of MPs or HMs include pollution load index (PLI) [25], land accumulative index (Igeo) [26], and potential ecological risk index (RI) [27]. However, these methods fail to consider the physical and chemical coupling effects and potential synergistic toxicity between MPs and HMs, which may lead to a significant underestimation of the true risk of composite pollution. Although Li et al. [28] proposed the first two-dimensional comprehensive index (TPI) model for evaluating MPs-HMs composite pollution, the model only considered the morphology of MPs and polymer toxicity without fully accounting for other key occurrence characteristics. Therefore, this study investigated the coexistence characteristics and enrichment mechanisms of MPs and HMs in sediments by comprehensively considering their interactions. Furthermore, the Multi Feature Potential Ecological Risk Index (MPERI) model was refined by integrating multiple intrinsic properties of MPs, including particle size, morphology, color, and polymer type, to achieve a more comprehensive and accurate assessment of the ecological risks posed by MPs-HMs composites in sedimentary environments. This study aimed to (1) elucidate the spatial distribution characteristics of MPs and HMs in the sediment of Daihai Lake; (2) assess the potential ecological risks of MPs, HMs, and their different forms in sediments; and (3) explore the potential mechanism of MPs as an emerging environmental medium in the migration and transformation of HMs.

## 2. Materials and Methods

### 2.1. Overview of the Research Area

**Figure 1:**
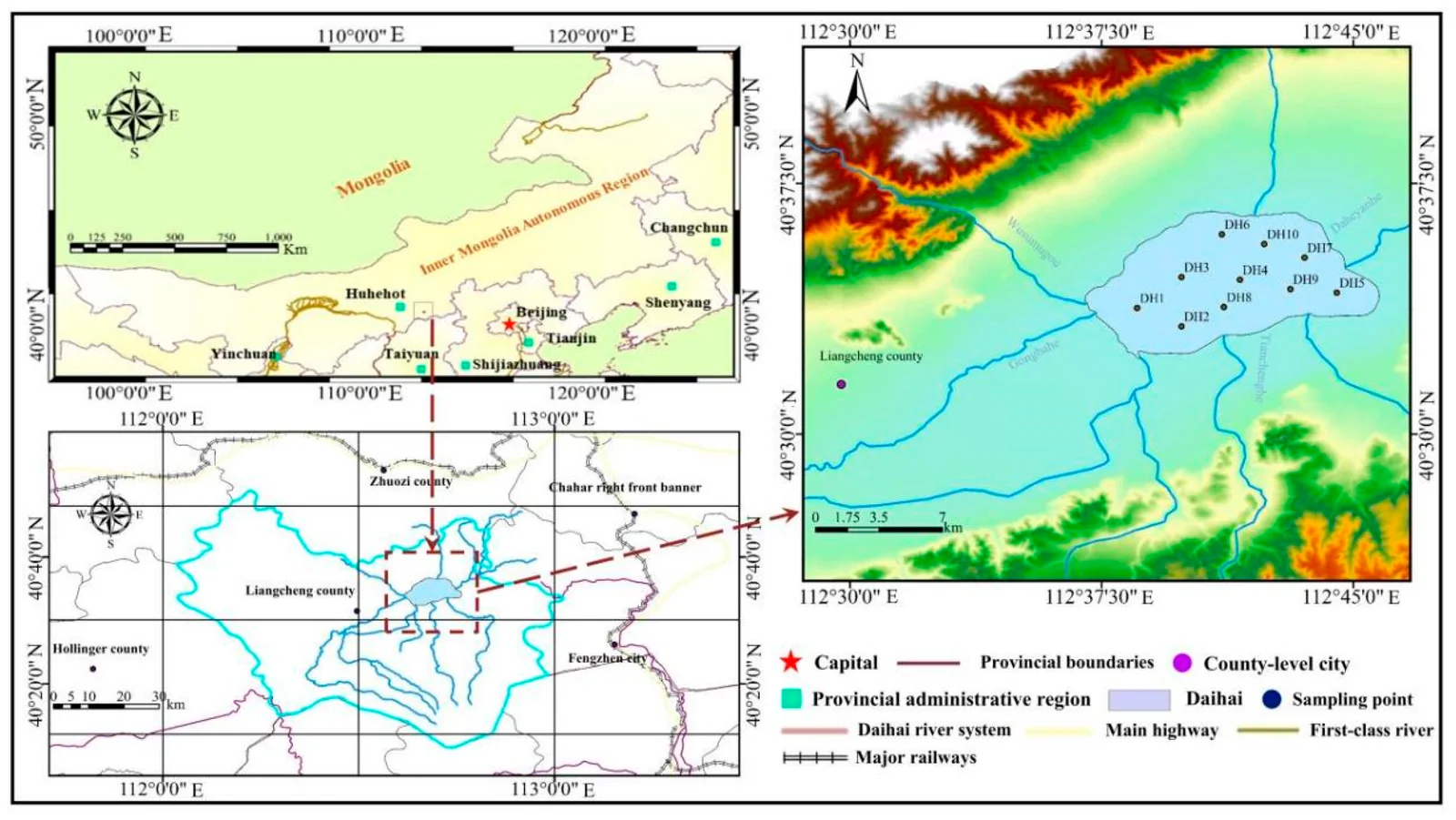
Sampling points in the study area.

Daihai Lake (40°29′7″–40°37′6″ N, 112°33′31″–112°46′40″ E) is located in Liangcheng County, Ulanqab City, Inner Mongolia Autonomous Region (Figure 1). It is the third largest inland lake in Inner Mongolia Autonomous Region, China, playing important roles in various fields such as ecology, economy, and culture. It is also a typical inland plateau type tail lake in the region, with the main supply system consisting of three seasonal inland rivers: Gongba River, Wuhao River, and Tiancheng River. Surface evaporation is the only discharge pathway, and the lake area, water storage capacity, and pollutant concentration are significantly affected by precipitation, groundwater, and watershed topography. Affected by both climate change and human production activities, the area of lakes is shrinking, and the process of water quality salinization continues to intensify. The Daihai Lake basin is characterized by strong evaporation, limited precipitation (annual average: 350–450 mm), and insufficient water replenishment, which mainly depends on groundwater and seasonal rivers with unstable streamflow. These conditions have caused continuous lake shrinkage, resulting in the concentration and accumulation of pollutants.

### 2.2. Sampling Sites

Based on the spatial grid distribution of Daihai Lake, the locations of inflowing rivers, population density, cultivated land, and industrial activities along the shoreline, a total of 10 sediment sampling sites were selected using satellite images, and samples were collected during the summer (June to August 2024). At each site, three replicate sediment samples were collected and homogenized to form a composite sample. All subsequent laboratory analyses were performed in triplicate for each composite sample. The sampling locations were recorded using a GPS device, and the sampling sites are shown in Figure 1. The collected samples were sealed with aluminum foil and transported to the laboratory, where they were stored at 4 °C prior to analysis.

### 2.3. Sample Processing and Detection Observation

#### 2.3.1. MPs Processing and Identification

The retrieved sediment samples were dried in a 50 °C oven for 24 h to avoid the MPs from being degraded at high temperatures and affecting the accuracy of the analysis. After drying, visible impurities such as gravel and plant residues in the soil matrix were manually removed. Given that some polymers, such as polyvinyl chloride (PVC, density ≈ 1.38 g·cm^−3^) and polyethylene terephthalate (PET, density ≈ 1.40 g·cm^−3^), have higher densities than a saturated NaCl solution (ρ ≈ 1.2 g·cm^−3^), using NaCl would lead to incomplete MPs recovery and introduce significant bias, even though NaCl has advantages such as low cost and environmental friendliness [29]. To ensure the coverage of the full density range of common MPs in the environment, this study selected saturated sodium iodide (NaI) solution (ρ = 1.8 g·cm^−3^ at 25 °C) as the flotation medium. In the experiment, 10.00 g of dried sediment was weighed, added to a saturated NaI solution, mixed thoroughly, and placed in a constant temperature shaker. The mixture was shaken at 25 °C for 12 h, and then allowed to stand for 12 h to complete the flotation separation of MPs. The supernatant was then filtered through a 300 mesh stainless steel sieve, and the sample on the sieve was transferred to a beaker using saturated NaI solution for flotation. The experiment was repeated three times to ensure accuracy. The surface supernatant in the beaker was filtered through a 300 mesh stainless steel sieve, and the MPs sample on the sieve was digested using Fenton reagent [30] on a constant temperature shaker at 40 °C and 120 rpm for 12 h. Afterwards, filtered the solution through a 0.45 μm pore size glass fiber filter membrane (Whatman, Marlborough, MA, USA), let it dry naturally, and stored it in a glass dish for testing.

MPs retained on the glass fiber filter membranes were photographed using a Leica stereomicroscope (M165C, Leica, Berlin, Germany). Their particle size and morphology were subsequently analyzed using LAS X software (Version 5.3.3). This study categorized MPs shapes into fiber, fragment, and film forms. The particle sizes were classified into three intervals: <0.25 mm, 0.25–0.50 mm, and >0.50 mm. Representative images of MPs are presented in Figure S1. The polymer composition of MPs was identified using Fourier transform infrared spectroscopy (FTIR; Nicolet iN10, Thermo Fisher Scientific, Waltham, MA, USA) over a wavelength range of 4000–400 cm^−1^. The polymer composition of MPs was identified by comparison with a standard plastic spectral library, and spectra with a matching degree greater than 70% were considered acceptable for identification [31].

#### 2.3.2. Testing of HMs Samples

The dried sediment was ground using agate mortar and sieved through a 100 mesh sieve. The sediment samples were digested according to the “HJ832-2017 Soil and Sediments Total Metal Element Digestion Microwave Assisted Acid Digestion Method” issued by the Ministry of Ecology and Environmental Protection of China [32]. The chemical forms of HMs in dry sediment were extracted using the four-step continuous extraction method published by the European Community Standard Reference Material Office (BCR) [33]. HMs were formated into four operationally defined forms: acid-extractable (F1), reducible (F2), oxidizable (F3), and residual (F4) [34]. The concentrations of each form were determined using inductively coupled plasma mass spectrometry (ICP-MS; NexION 1000/2000, PerkinElmer, Shelton, CT, USA). The calibration curves showed excellent linearity (R^2^ = 0.9997). Detailed experimental procedures are provided in Supplementary Text S1.

### 2.4. Risk Assessment

#### 2.4.1. Pollution Load Index

Assessing the risk of MPs in water using PLI model [35]. This method was first used to evaluate the pollution level in estuaries and has been widely promoted for risk assessment of MPs. The calculation is shown in Equations (1)–(3).

(1) $$\mathrm{PLI}=\sqrt{CF_{i}},$$

(2) $$CF_{i}=C_{i}/C_{\mathrm{oi}},$$

(3) $$\mathrm{PL}I_{\mathrm{zone}}=\sqrt[{n}]{\mathrm{PL}I_{1}\times \mathrm{PL}I_{2}\times \mathrm{PL}I_{3}\times \cdots \times \mathrm{PL}I_{n}},$$

where CF_i_ is the abundance factor of MPs, C_i_ is the detection abundance of MPs, n·g^−1^, C_oi_ serves as a reference for the average abundance of MPs in similar study areas [36], but Xu et al. [37] pointed out that C_oi_ does not affect its use. Supplementary Table S1 divides the PLI into four levels.

#### 2.4.2. Risk Characterization Ratio (RCR)

The RCR evaluates the degree of ecological pollution caused by MPs in the study area based on their abundance and polymer composition, and is divided into four levels (Supplementary Table S2) [38]. The calculation formula is shown in Equations (4)–(7).

(4) $$Z_{i}=k\;\times \;S_{i},$$

(5) $$H=\sum P_{i}\times Z_{i},$$

(6) $${\mathrm{MEC}}_{Z}=\mathrm{MEC}\;\times \;H,$$

(7) $$\mathrm{RC}R\;=\frac{{\mathrm{MEC}}_{Z}}{\mathrm{PNEC}},$$

where S_i_ represents the hazard index of plastic polymer i, K is the adjustment coefficient, with a value of 1/15 [39], and Z_i_ is the adjusted hazard index for plastic polymer i (Supplementary Table S3). This study used polymer hazard index data to calculate the polymer risk index (H) caused by MPs, which was used as an indicator for classification [40,41], (Supplementary Table S4). P_i_ is the proportion of the i-th plastic polymer at each point, MEC represents the abundance of MPs at each sampling point, n·kg^−1^, and PNEC is the background value of MPs abundance (540 n·kg^−1^).

#### 2.4.3. Potential Ecological Risk Index

Hakanson proposed using the RI method to evaluate HMs pollution and its ecological hazards. This method comprehensively considers the ecological, environmental, and toxicological effects of harmful substances, and can comprehensively reflect the impact of harmful substances on the ecological environment [42]. The calculation is shown in Equations (8) and (9).

(8) $$E_{r}^{i}=T_{r}^{i}\;\times \;(\frac{C_{i}}{C_{0i}}),$$

(9) $$\mathrm{RI}=\sum_{i=1}^{m}E_{r}^{i},$$

where C_i_ and C_0i_ are the measured and reference values of HMs i, $T_{r}^{i}$ is the toxicity coefficient of HMs i, $E_{r}^{i}$ is the potential ecological hazard index of HMs i, RI is the comprehensive potential ecological risk index of various HMs in the study area, and the risk assessment is shown in Supplementary Table S5.

#### 2.4.4. Ratio of Secondary Phase to the Primary Phase

The ratio of secondary phase to primary phase (RSP) is another ecological risk assessment method based on heavy metal speciation [43], and its calculation is given in Equation (10).

(10) $$\mathrm{RSP}={\;M}_{\sec}/M_{\mathrm{prim}},$$

where M_sec_ represents the concentration of HMs secondary phases (F1 + F2 + F3) in sediment, and M_prim_ represents the concentration of residual HMs primary phases (F4) in sediment. Among them, RSP < 1, 1 < RSP < 2, 2 < RSP < 3, and RSP > 3 respectively indicate no pollution, mild pollution, moderate pollution, and severe pollution.

#### 2.4.5. Risk Assessment Coding Method

The RAC is mainly used to evaluate the ecological risk of HMs based on the proportion of form F1 state concentration of HMs to the total amount of elements [44,45]. The calculation is shown in Equation (11).

(11) $$\mathrm{RAC}=\frac{C_{F1}}{C_{T}}\times 100\%,$$

where C_F1_ is the concentration of F1 + F2 + F3 + F4, mg·kg^−1^, and C_T_ is the total amount of HMs, mg·kg^−1^. RAC < 1%, 1% ≤ Rac < 10%, 10% ≤ Rac < 30%, 30% ≤ Rac < 50%, and RAC ≥ 50% respectively indicate no risk, low risk, medium risk, high risk, and extremely high risk.

#### 2.4.6. MPs-HMs Joint Risk Assessment

The MPERI method incorporates multidimensional features such as the type, toxicity effects, color, particle size, and shape of MPs into the evaluation system to comprehensively assess the ecological risks of MPs [46]. This method has strong comprehensiveness and is suitable for complex pollution systems, avoiding treating multiple pollutants as prominent single pollutants and affecting the evaluation results. The calculation is shown in Equations (12)–(15).

(12) $$T_{{\mathrm{color}}_{i},{\mathrm{shape}}_{i}{,\mathrm{polymer}}_{i}=}\sum_{i=1}^{n}P_{{\mathrm{shape}}_{n},{\mathrm{color}}_{n}{,\mathrm{polymer}}_{i}}\times S_{{\mathrm{shape}}_{n},{\mathrm{color}}_{n}{,\mathrm{polymer}}_{i}},$$

(13) $${\mathrm{Ti}}_{m}=\sum_{i=1}^{n}T_{\mathrm{polymer}}\times (0.2\;\times \;T_{\mathrm{color}}+0.8\;\times \;T_{\mathrm{shape}})P_{\mathrm{size}},$$

(14) $$\mathrm{MPERI}=\mathrm{PLI}\;\times \;{\mathrm{Ti}}_{m},$$

(15) $$\mathrm{TPI}\sqrt{0.5\;\times \;{\mathrm{MAX}(\mathrm{MPERI},\mathrm{RI})}^{2}+0.5\;\times \;{\mathrm{AVERAGE}(\mathrm{MPERI}+\mathrm{RI})}^{2}},$$

where Ti_m_ is calculated as the new toxicity index based on the polymer hazard index (T_polymer_), shape hazard index (T_shape_), color hazard index (T_color_), and the percentage of small MPs (P_size_). T_polymer_ is the same as RCR, where P_i_ represents the proportion of different characteristics of MPs in the sediment at the sampling site, S_i_ represents the hazard score corresponding to different characteristics of MPs, and the MPs characteristic score is shown in Supplementary Tables S6 and S7.

### 2.5. Data Analysis

Before conducting statistical analysis, this study first evaluated the normality (shapiro wilk test) and homogeneity of variance (levene test) of the data. The data analysis was mainly completed through SPSS 25.0 and R language (RStudio integrated development environment, R version 4.2.2). Given the non normality of data distribution and potential non-linear relationships between variables, spearman rank correlation analysis and mantel test were used to evaluate the association between MPs and HMs. Inter group differences were identified through one-way ANOVA combined with LSD multiple comparison test (significance level set at *p* < 0.05). Descriptive statistics are expressed as mean ± standard deviation and extreme outliers identified based on box plot criteria are removed before calculation. The chart was created using Origin 2021 and R (packages such as ggplot2, dplyr, vegan, etc.), and the spatial sampling distribution map was generated using ArcGIS 10.2.

### 2.6. Quality Allegations

To minimize contamination by airborne microplastics (MPs), cotton laboratory clothing was worn throughout the experimental procedures. All containers used for MP analysis were made of glass, and plastic materials were avoided whenever possible. Before use, all containers were rinsed three times with ultrapure water and dried. Quality assurance (QA) and quality control (QC) were performed by simultaneously analyzing duplicate samples, reagent blanks, and certified reference materials together with the field samples. The national soil certified reference material GSS-8 (GBW-07408), blank samples, and parallel samples were used for quality control. In addition, three parallel samples and two procedural blanks were included in each batch of analyses, and no MPs or HMs were detected in the blank samples.

To evaluate the accuracy and reproducibility of the sample pretreatment process [47], this study used polyethylene terephthalate (PET, density 1.38 g·cm^−3^) and polypropylene (PP, 0.91 g·cm^−3^) MPs standards for recovery experiments. The standard particles used were spherical with a nominal particle size of 100 μm (purchased from Triumph Plastics Technology Co., Ltd., Shenzhen, China). A known amount of standard particles was added to ultrapure water, and the pretreatment process was then performed in complete accordance with the actual sediment sample. The recovered particles were quantitatively counted using a stereomicroscope, and their polymer type was verified by FTIR. During the three-month study period, method performance was evaluated monthly, with three independent rounds of experiments completed. Each round had three parallel replicates to examine intra-batch and inter-batch variability. The recoveries and relative standard deviations (RSDs) of each polymer were calculated accordingly. The results showed that the average recoveries (±standard deviation) of PET and PP MPs were (97.1 ± 4.2)% and (99.7 ± 3.7)%, respectively, with corresponding RSDs of 4.72% and 3.18%. These results indicated that the pretreatment method established in this study had high recovery efficiency for typical MPs with different densities, and the analytical process exhibited good precision and excellent reproducibility, making it suitable for the quantitative analysis of MPs in sediments.

To prevent potential contamination, all glass bottles were pre-treated by immersion in 10% nitric acid (HNO_3_), followed by rinsing with distilled and deionized water, and oven-drying prior to use. Collected samples were immediately sealed with aluminum foil and transported to the laboratory at 4 °C. A rigorous quality assurance and quality control (QA/QC) protocol was implemented throughout the process. During field sampling, three field blanks (1 L deionized water) were placed alongside the sampling stations to monitor environmental contamination. Analysis showed that black PP particles were present in field blanks, but their abundance was <10% of the total MPs found in actual samples, indicating acceptable background levels [48]. In the laboratory, two procedural blanks were processed with each batch of samples. The final MP counts in sediment and water were corrected by subtracting the average number of particles found in the corresponding blanks to eliminate background interference.

## 3. Results and Discussion

### 3.1. Spatial Distribution Characteristics of MPs in Sediments

#### 3.1.1. Total Amount and Horizontal Spatial Distribution Characteristics of MPs

**Figure 2:**
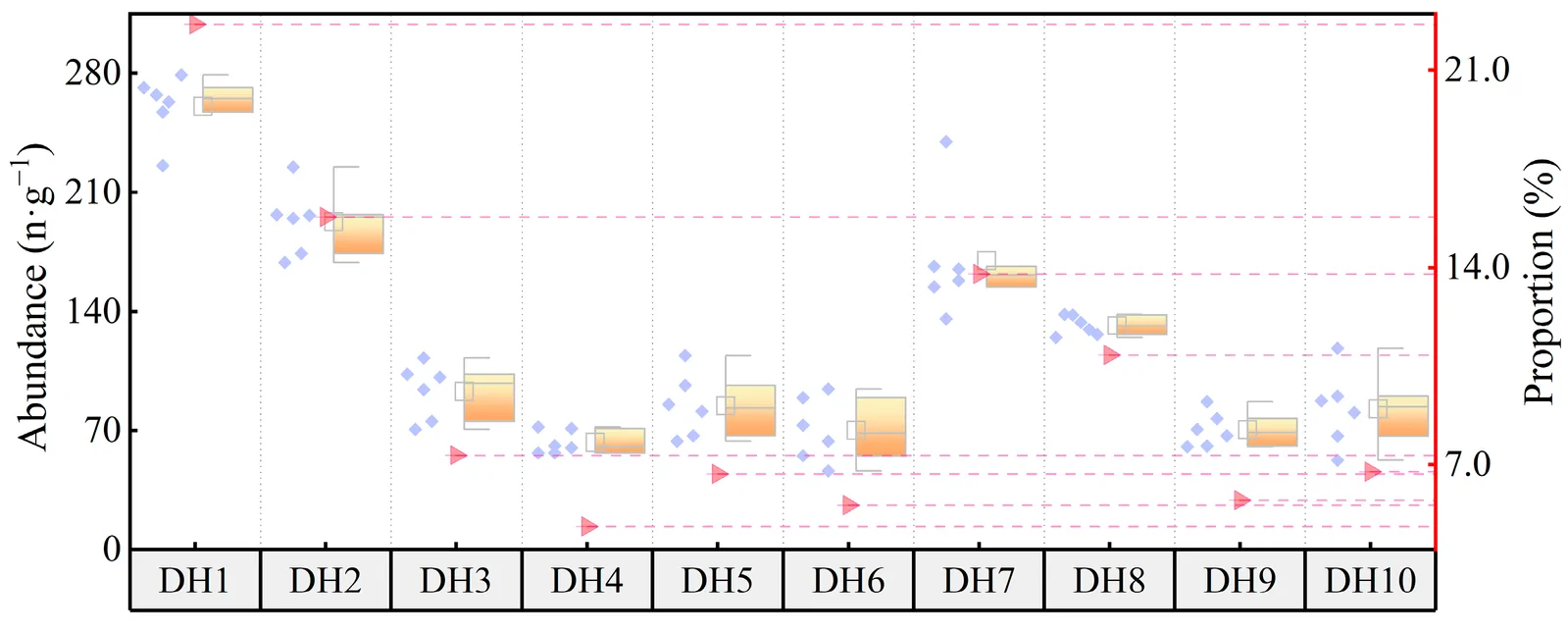
Horizontal distribution characteristics of MPs.

The abundance of MPs in the sediments of Daihai ranged from 7 to 27 n·g^−1^ (Figure 2), with an average value of 13 ± 5 n·g^−1^, which was generally higher than that reported in other regions (Supplementary Table S7). Based on the sampling sites shown in Figure 1, spatial heterogeneity in the distribution of MPs was observed, with an overall decreasing trend in abundance from southwest to northeast. Among all sampling sites, DH1 and DH2 exhibited the highest MP abundances, accounting for more than 15.00% of the total. As the sampling sites approached residential areas along the lakeshore, the input of MPs into the lake gradually increased due to intensified human activities. Yin et al. [49] also pointed out that human factors are one of the main reasons affecting the abundance of MPs. Influenced by surrounding farmland and transportation factors, MPs wrapped in soil dust are brought into the lake by wind, resulting in an increase in abundance. The depth of Daihai water decreases from the center of the lake to the shore. Due to the longer time required for MPs suspended near the center of the lake to settle, they are more susceptible to factors such as wind or water flow disturbances during the settling period and are unable to settle. The results showed that the proportion of MPs in the center of the lake fluctuated around 10%, whereas the abundance of MPs along the shoreline in uninhabited areas decreased significantly, with the proportion fluctuating around 7%. DH5, located at the easternmost part of the lake, was influenced by groundwater recharge and hydrodynamic disturbance, which promoted sediment resuspension and made the settlement of MPs more difficult, resulting in a further decrease in the proportion of MPs in the sediments of DH5 [50].

#### 3.1.2. Distribution and Spatial Differences in MPs Occurrence Forms

**Figure 3:**
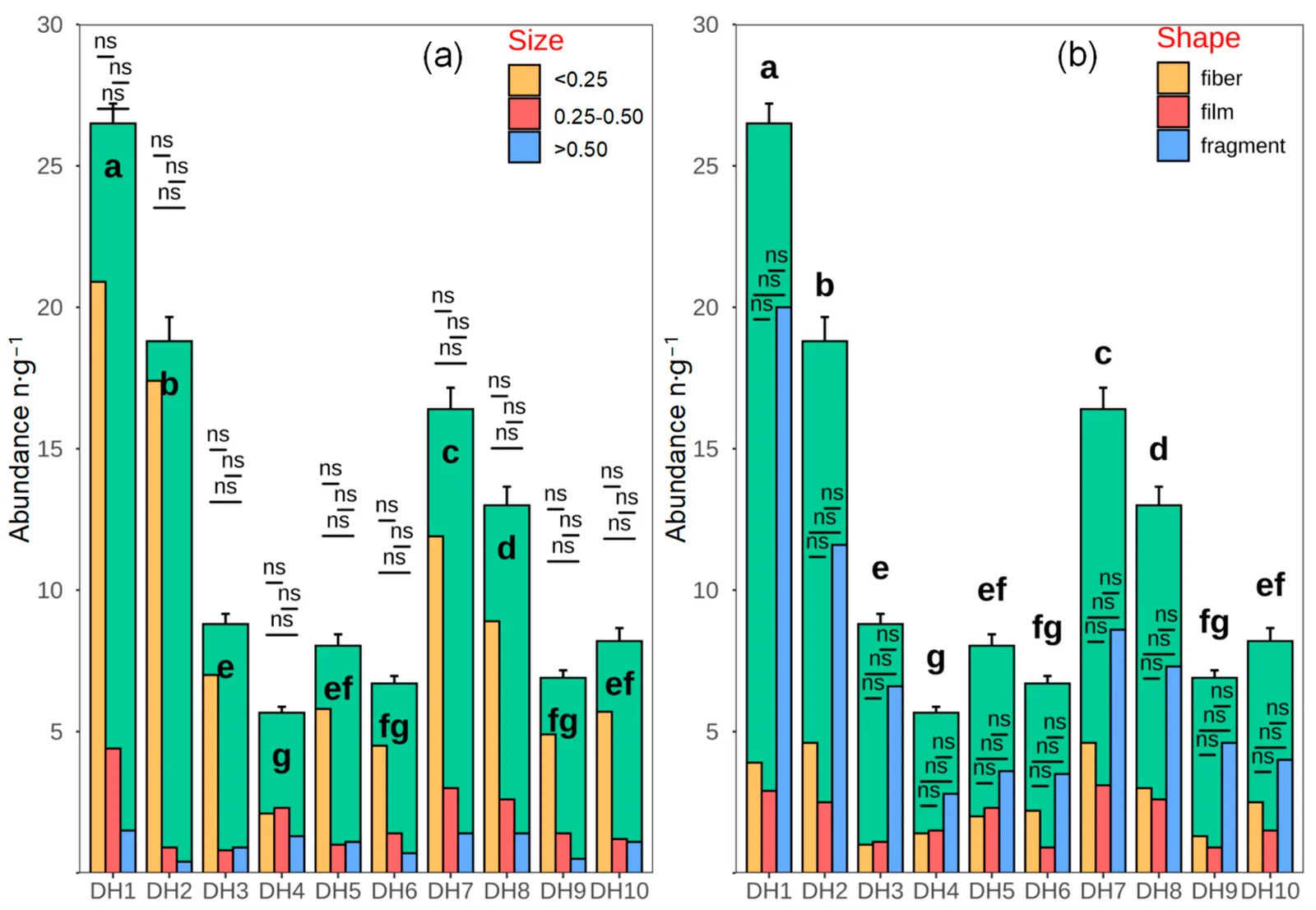
Horizontal distribution of MPs particle size: (**a**) abundance of MPs size and (**b**) abundance of MPs shapes. Different lowercase letters (a, b, c…) indicate significant differences between groups (*p* < 0.05), while sharing the same letter indicates no significant differences.

This study found through multiple sampling and monitoring that the closer the point is to the center of the lake, the more stable the abundance of MPs, indicating that it is less disturbed by human and natural conditions. Experiments revealed that MPs with a size less than 0.25 mm were predominant in the sediments, accounting for an average of over 75% (Figure 3a), followed by MPs with a size between 0.25 mm and 0.50 mm, accounting for 14%. MPs with particle sizes greater than 0.50 mm were the least abundant, indicating that smaller MPs were more likely to accumulate in the sediments. This pattern may be attributed to the prolonged residence time of MPs in sediments, where continuous biological abrasion and physicochemical degradation can promote their fragmentation into smaller-sized microplastics and, potentially, nanoplastics. Supplementary Figure S2 showed that the abundance of MPs smaller than 0.25 mm was the highest along the western shore and gradually decreased eastward, whereas MPs larger than 0.50 mm radiated outward from the lake center, reaching the highest abundance along the eastern and western shores. Our intergroup significance tests for different particle sizes at each sampling point all showed “ns” (*p* > 0.05), indicating that there was no statistically significant difference in the abundance of MPs among the particle size groups within the same sampling point. This may be related to the high heterogeneity of the samples or the natural variability of the particle size distribution.

Fragmented MPs were the dominant type in the sediments (Figure 3b), accounting for more than 60%, followed by fibers (21%) and films (15%), which were consistent with the findings of Huang et al. [51]. The characteristics of MPs in different lakes depend on lake type, function, and the intensity of surrounding human activities. The abundance of fragmented MPs showed a decreasing trend from east to west, whereas the abundance of film MPs decreased from the lakeshore toward the lake center. However, the highest abundance of all three MP shapes was observed along the western shore (Supplementary Figure S3). This pattern was likely attributed to intensive human activities, such as fishing and tourism, along the lakeshore, which increased the input of fragmented MPs. In addition, MPs originating from surrounding farmland and residential areas also entered the lake through atmospheric deposition and rainfall runoff. The abundance of film MPs remained relatively stable, accounting for less than 15%, whereas fibrous MPs fluctuated between 20% and 30%. Fragmented MPs were not only the most abundant shape but also exhibited the greatest spatial variability, accounting for 40–70% of the total MPs. Furthermore, intergroup significance tests were conducted for different MP shapes at each sampling site, and the results showed no significant differences among the shape groups within the same sampling site (*p* > 0.05).

The proportion of MPs colors in the sediment of Daihai is shown in Supplementary Figure S4, with black being the main color (22%), followed by red (19%), white (16%), blue (16%), green (15%), and yellow (13%). The dominance of black MPs indicated that they mainly originated from urban runoff and human activities. Black MPs were often associated with the use of agricultural plastic films and fishery waste, such as discarded fishing nets and ropes, which was consistent with the important role of local fisheries in regional economic activities [52]. In contrast, the relatively high proportions of red, blue, and white MPs reflected the widespread input of plastic debris from consumer products, including packaging materials, household containers, and synthetic textiles.

#### 3.1.3. Identification of MPs Composition

**Figure 4:**
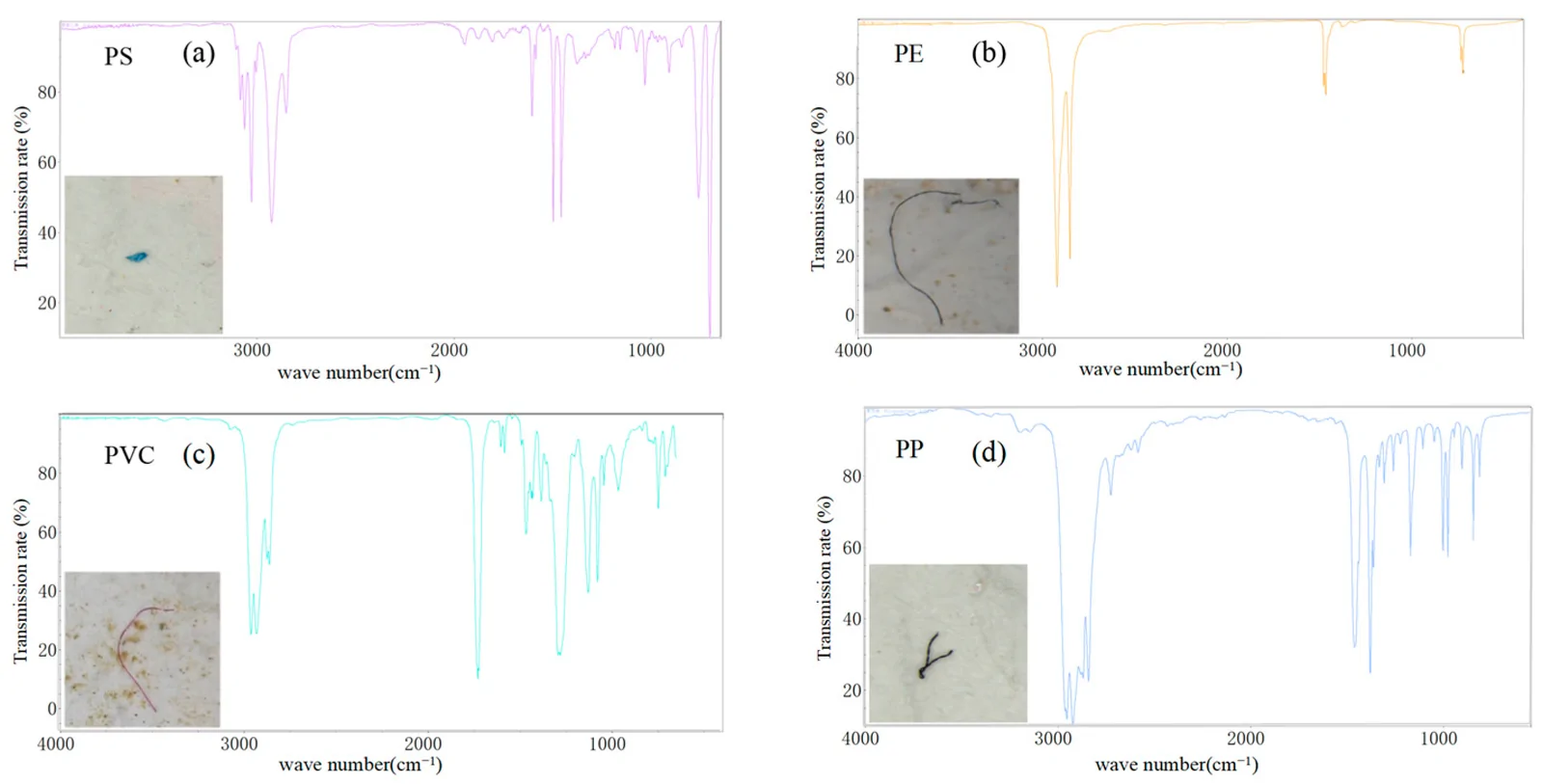
FTIR of polymer composition: (**a**) PS, (**b**) PE, (**c**) PVC, (**d**) PP.

The polymer composition of MPs was identified by FTIR, and the results are shown in Figure 4 and Supplementary Text S1. PET was detected in all sediment samples, with a relative abundance ranging from 10% to 46%, averaging over 30%. Polypropylene (PP) had an average abundance of 25%, followed by polyethylene (PE) with an average abundance of 20%. In contrast, polyvinyl chloride (PVC) and polystyrene (PS) had lower detection rates, averaging 12% and 8%, respectively. To ensure the representativeness of the samples and minimize selection bias, MPs were randomly selected from each filter membrane for polymer composition identification, thereby covering the morphological and compositional diversity of MPs in the samples. Specifically, for sediment samples from each month, we randomly selected 450 identifiable MP particles for FTIR analysis (a total of 1350 particles). Of these, 1184 particles (88%) were successfully identified as six different polymer types (Figure 4). The high abundance of PET and PP in sediments is closely related to their widespread use in the Daihai Basin. For example, PET is the main raw material for synthetic fiber textiles, and fiber shedding during clothing washing and textile production is an important source of PET MPs in the environment [53]. On the other hand, plastic products used extensively in agricultural activities, such as mulch film, woven bags, and fruit and vegetable packaging films, are mainly composed of PP and PE [54]. In addition, PP and PE, as typical thermoplastic resins, are widely used in the fishery field due to their excellent flexibility and durability, such as the manufacture of fishing nets, fishing lines and other equipment [55]. Given that fishery is an important industry in the Daihai area, physical wear and tear of fishing gear during use is also a key pathway for the continuous input of PP and PE MPs into the water.

### 3.2. Distribution Characteristics and Forms of HMs in Sediments

Supplementary Figure S5 shows the concentration changes of HMs in the sediment of the study area, with the average concentration ranking as follows: Mn (863 ± 78 mg·kg^−1^); Cr (124 ± 28 mg·kg^−1^); Zn (86 ± 17 mg·kg^−1^); Ni (43 ± 10 mg·kg^−1^); Cu (36 ± 0.1 mg·kg^−1^); Pb (23 ± 5 mg·kg^−1^); As (15 ± 4 mg·kg^−1^); and Cd (0.20 ± 0.04 mg·kg^−1^). As, Cu, and Pb mainly enter the environment through the combustion of fossil fuels, due to the presence of coal-fired power plants around Daihai, the exhaust gas generated from their combustion can be transported into lake sediments through atmospheric deposition pathways, including As, Cu, and Pb [56]. In contrast, Ni, Cr, and Cd mainly come from industrial emissions and agricultural waste [57]. It is worth noting that Daihai has both tourism and aquaculture functions. Frequent ship activities have led to rusting of the hull and peeling of anti-fouling paint, becoming an important source of Cu, Pb, Cr, Cd, and Zn pollution in sediments [58]. The coefficient of variation (CV) of HMs in sediments was ranked as follows: Cd > Cr > As > Ni > Zn > Mn > Cu > Pb (Supplementary Table S8), with Cd having the highest CV (Supplementary Table S9), indicating uneven spatial distribution, strong variability, and significant interference from human activities [59], suggesting that Cd may be a sensitive indicator factor for anthropogenic pollution in this lake.

This study systematically analyzed the different chemical forms of HMs in sediments. The effectiveness analysis of HMs can be found in Supplementary Text S2 and Figure S8, and the concentration distribution of each form is shown in Supplementary Figure S6. Among them, the F4 form concentration of Cr and Zn is very high, accounting for nearly 100%. The F4 form of Ni, Cu, As, and Pb shows a decrease in concentration compared to Cr and Zn, but still accounts for over 90.00%. The F4 form mainly occurred within the crystal lattices of primary and secondary silicate minerals. Consequently, it exhibited low mobility and bioavailability and was therefore difficult for benthic organisms to utilize, resulting in relatively limited environmental risk [60]. In contrast, the F1 form in the non-residual phase was the most sensitive to environmental changes and exhibited high mobility and bioavailability, making it more likely to be released into the environment and pose risks to aquatic organisms [61]. Cr and Zn were mainly present in the F2 form. Chrysochoou et al. [62] reported that the negative charge on the surfaces of clay minerals and hydrated oxides increased with increasing environmental pH, thereby promoting hydroxide formation of metal ions and increasing the proportion of the F2 form. However, the average pH value in the study area was always around 9.00. Under these environmental conditions, the adsorption and precipitation of Fe/Mn oxides play a dominant role. Therefore, most of the bioavailable Cu, Ni, As, and Pb occurred in the F2 form, indicating that these metals exhibited relatively low mobility and posed limited environmental risk under the prevailing conditions. However, when the redox potential decreased or under hypoxic conditions, the F2 form could be transformed into more bioavailable forms, thereby increasing the potential risk of secondary pollution [57,62]. Ni, Cu, As, and Pb mainly occurred in the sulfide-bound and F3 forms, and the F3 form was released only under strongly oxidizing conditions, further enhancing their stability.

### 3.3. Research on the Coexistence Characteristics and Enrichment Mechanism of MPs-HMs

#### 3.3.1. Correlation Testing and Cluster Analysis of MPs and HMs

**Figure 5:**
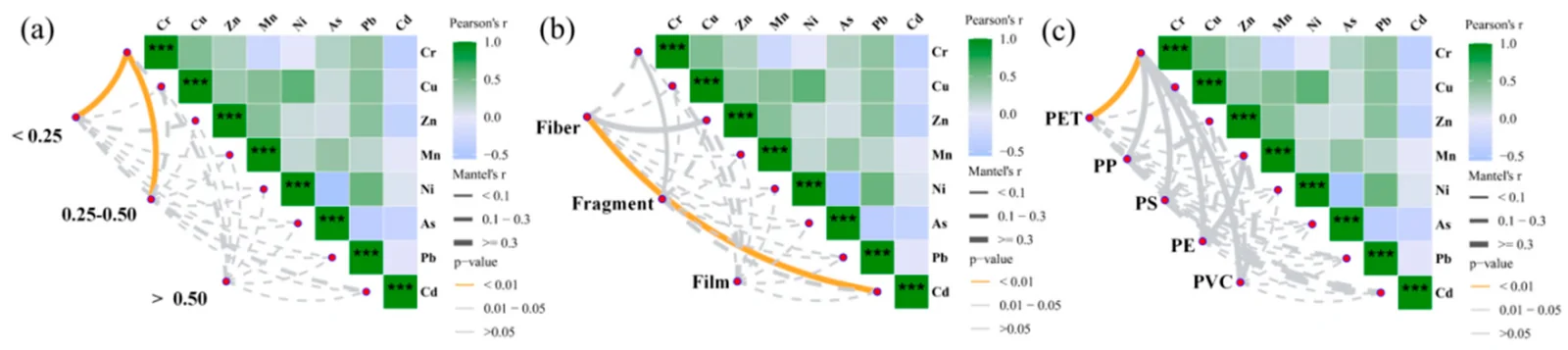
Mantel detection chart of MPs and HMs: (**a**) correlation between different sizes of MPs and HMs, (**b**) correlation between different shapes of MPs and HMs, and (**c**) correlation between different components of MPs and HMs, ***: *p* < 0.001.

The Mantel test showed (Figure 5) that MPs were correlated with HMs in the sediments. MPs with particle sizes of <0.25 mm and 0.25–0.50 mm were significantly correlated with Cr (*p* < 0.01), whereas fibrous MPs exhibited a strong correlation with Cd. The study also found varying degrees of correlation between different types of polymers and HMs, indicating that the properties of MPs may influence their interaction with HMs. The above results collectively reveal that the mutual adsorption effect between fibrous MPs with small sizes and large specific surface area and HMs is more prominent. It is worth noting that the sampling depth of this study was the surface sediment (5–10 cm), which is located in the oxidation-reduction transition zone and had limited oxygen permeation and a local reduction microenvironment. The presence of oxygen can accelerate the photo-oxidation and aging process of MPs, leading to surface cracks, pores, and irregular morphology, which can significantly increase their specific surface area. After aging under oxidative conditions, previous studies revealed that common polymers, such as PP and PE, could increase their specific surface areas by 4–6 times [63], thereby further enhancing their ability to chelate and enrich HMs. Correlation analysis also showed that Cd had a strong positive correlation with Cu and Pb, suggesting that these metals might have shared common pollution sources or been influenced by similar environmental factors [64,65]. In contrast, Cd was negatively correlated with As, Zn, and Cr, indicating that these elements might have competed for adsorption sites during their migration and transformation. To further analyze the coupling patterns among pollutants, cluster analysis was performed on the pollutant composition (Supplementary Figure S7). The clustering analysis results showed that pollutants were divided into three main categories. The first category included MPs and fragmented particles with particle sizes smaller than 0.25 mm, and their standardized data values were generally high, indicating that this component had high reactivity and environmental sensitivity and might preferentially adsorb HMs during migration. The second category included fibrous and film-like MPs, 0.25–0.50 mm particle size forms, and Mn, showing strong correlations, which suggested that they might have similar source characteristics or co-deposition behaviors. The third category consisted of MPs with particle sizes greater than 0.50 mm and other HMs except Mn. The correlation coefficients were mostly negative or close to zero, indicating that coarser particles had a weaker association with HMs or follow different migration pathways and environmental trends.

#### 3.3.2. Analysis of the Coexistence and Enrichment Mechanism of HMs on the Surface of MPs

MPs exhibit significant affinity for metal ions due to their high specific surface area and inherent hydrophobicity. This characteristic promotes the rapid and widespread enrichment of HMs on their surfaces, making them important carriers for pollutant migration [66], thereby exacerbating the ecological toxicity effects of composite pollution in water environments. The interaction mechanism between MPs and HMs is multifaceted and complex. Firstly, the aging process and ultraviolet radiation can induce surface oxidation of MPs, generating polar functional groups such as carboxyl, hydroxyl, and carbonyl [67], and introducing structural defects to enhance surface charge density, thereby promoting electrostatic attraction and coordination complexation reactions. Secondly, cation exchange and physical adsorption also participate in the binding process of metals. In addition, in near neutral environments, inorganic minerals such as iron/manganese (oxygen) hydroxides or calcium carbonate are prone to deposit on the surface of MPs, leading to surface modification of particles and the formation of additional metal binding active sites, further enhancing the adsorption capacity of MPs. It is particularly crucial that the formation of biofilm on the surface of MPs introduces a bio-mediated pathway. Extracellular polymers (EPS) in the biofilm are rich in functional groups such as carboxyl, amino, and phenolic hydroxyl groups, which can act as potent ligands to coordinate with metal ions, significantly altering surface charge characteristics and adsorption kinetics [21]. At the same time, organic matter in sediments can act as electron acceptors for microbial metabolism, driving the valence state transformation of HMs through microbially mediated redox reactions, indirectly affecting their mobility and bioavailability [68]. Moreover, MPs could directly influence the transformation of different HMs forms in sediments [69]. This effect was mainly attributed to their high hydrophobicity and large specific surface areas, which enhanced the interactions between MPs and HMs and consequently promoted the coupling processes between them. The non-specific chelation reaction reduces the bioavailability of HMs, thereby promoting the transformation of HMs from bioavailable states to stable organically bound states [70,71].

Unlike aquatic environments, MPs in sediments are not easily resuspended into overlying water bodies, but tend to remain and accumulate for a long time. On the one hand, they receive MPs input from water sedimentation, and on the other hand, they are continuously eroded by microorganisms in sediments. This biological degradation and physical wear process will further change the surface morphology and chemical properties of MPs, as mentioned earlier, thereby enhancing their interaction with HMs. It is worth noting that during ecological water replenishment, groundwater–lake water exchange, or hydrodynamic disturbances, previously immobilized HMs could be remobilized into the water column due to environmental changes, such as fluctuations in Eh and pH. This process alters their chemical speciation and release kinetics, thereby increasing the complexity and uncertainty of composite water pollution.

### 3.4. Analysis of Pollution Risk Assessment on Ecological Environment

#### 3.4.1. HMs Risk Assessment

**Figure 6:**
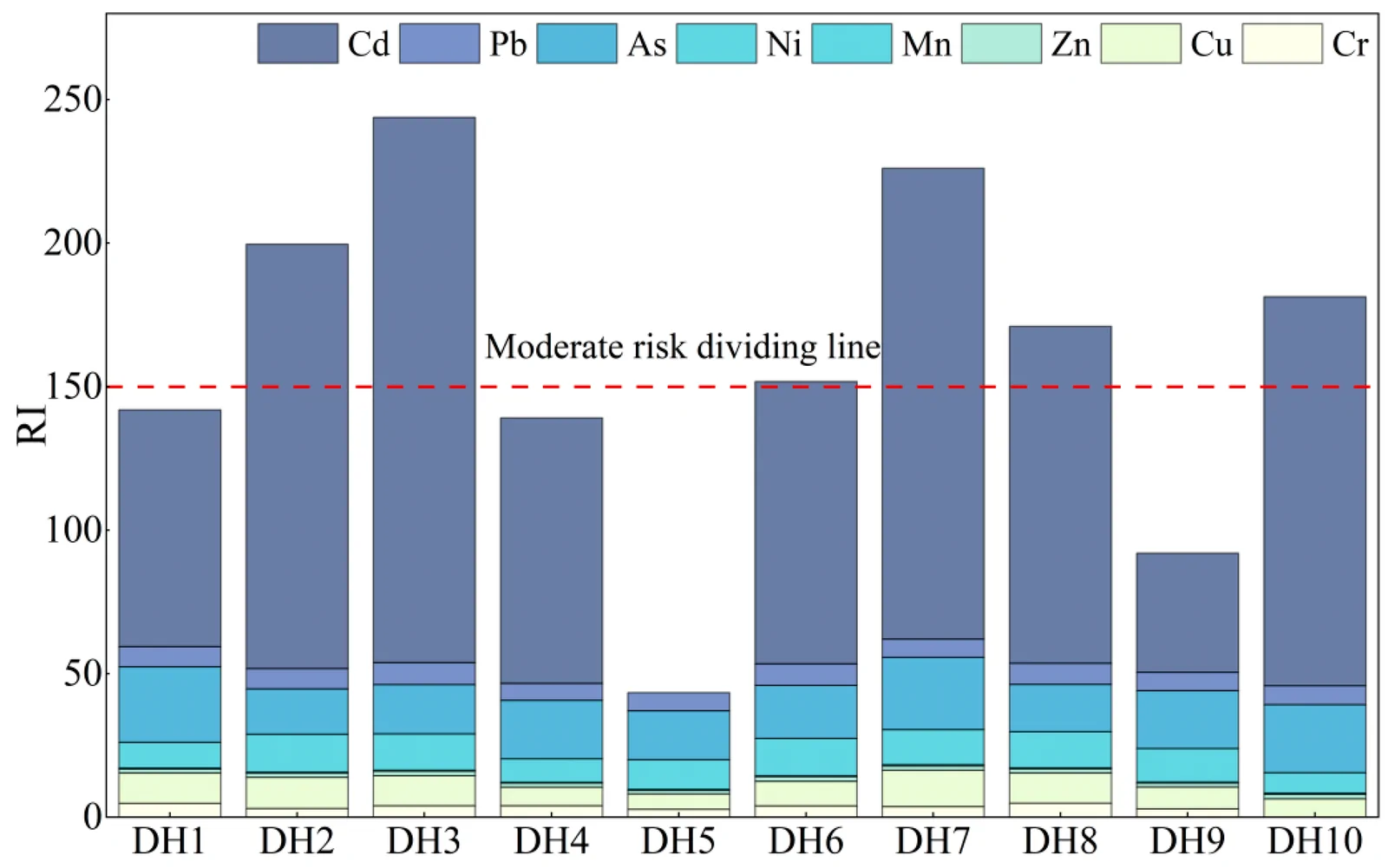
Potential ecological risks of HMs.

The ecological risk values of HMs in sediments were evaluated using the potential ecological risk assessment method (Figure 6). The contributions of the eight HMs to ecological risk were ranked as follows: Cd > As > Ni > Cu > Pb > Cr > Zn > Mn. According to the RI values, three sites exhibited high ecological risks associated with Cd, whereas the remaining sites showed relatively lower risks. The potential ecological risk indices of metals other than Cd in the sediments were all at low levels, highlighting the necessity of prioritizing Cd management in the study area. The RI values of sediments in the study area ranged from 43 to 244, with an average value of 159.34, indicating a moderate ecological risk level. Among the sampling sites, six showed moderate risks, with DH3, DH7, and DH10 exhibiting the highest comprehensive potential risks. Cd was the major contributor (45–78%), which was attributed to its high toxicity. DH4 and DH5 exhibited the lowest ecological risks, possibly due to their locations near the lake center and away from urban areas.

**Figure 7:**
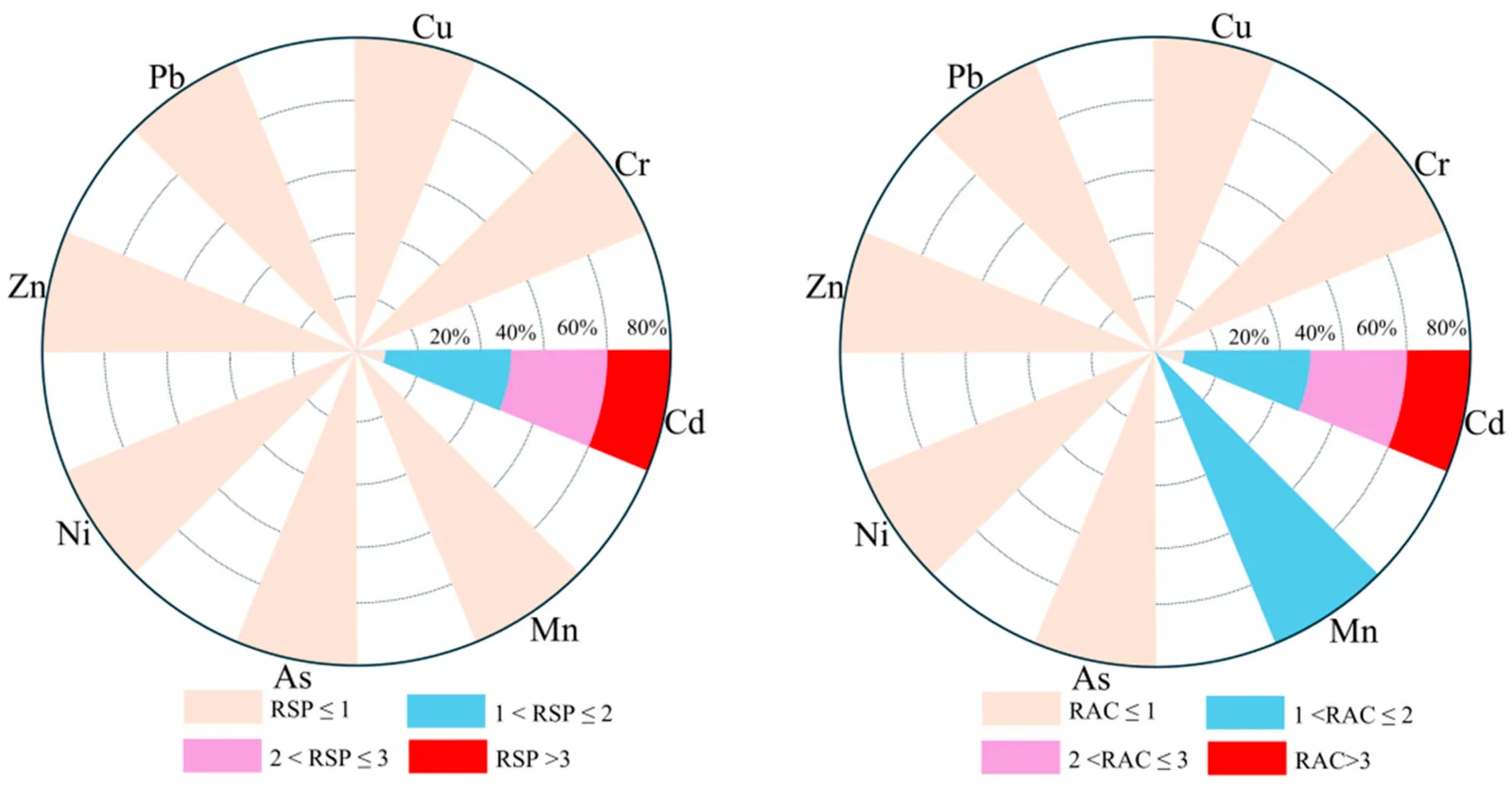
HMs form risk assessment.

This study analyzed the chemical speciation characteristics of HMs in sediments from the perspectives of RAC and RSP to assess the ecological risk to Daihai (Figure 7). Among all sampling sites, Cd mainly occurred in the F1 form, with only three sites showing low risk, whereas the remaining sites exhibited high or extremely high risks. This indicated that Cd in the sediments of the study area had high bioavailability, which was consistent with the findings of Zhu et al. [72]. In contrast, the five elements, including Cr and Cu, exhibited very low RAC values, indicating their limited bioavailability. The RSP results showed that among the eight HMs pollutants, Cd had the highest RSP value, with an average of 2.64. A total of two heavily polluted sites were found, with a relatively high degree of pollution. All elements except Cd were non-polluted. The results of RAC and RSP demonstrated that Cd in sediments posed the greatest ecological risk. Cd was a potent nephrotoxin and a primary carcinogen that could accumulate in the human body through the food chain, causing significant health risks [73]. The high proportions of its F1 and F2 forms indicated the necessity for further consideration of Cd management in water quality management.

#### 3.4.2. Risk Assessment and Analysis of MPs

**Figure 8:**
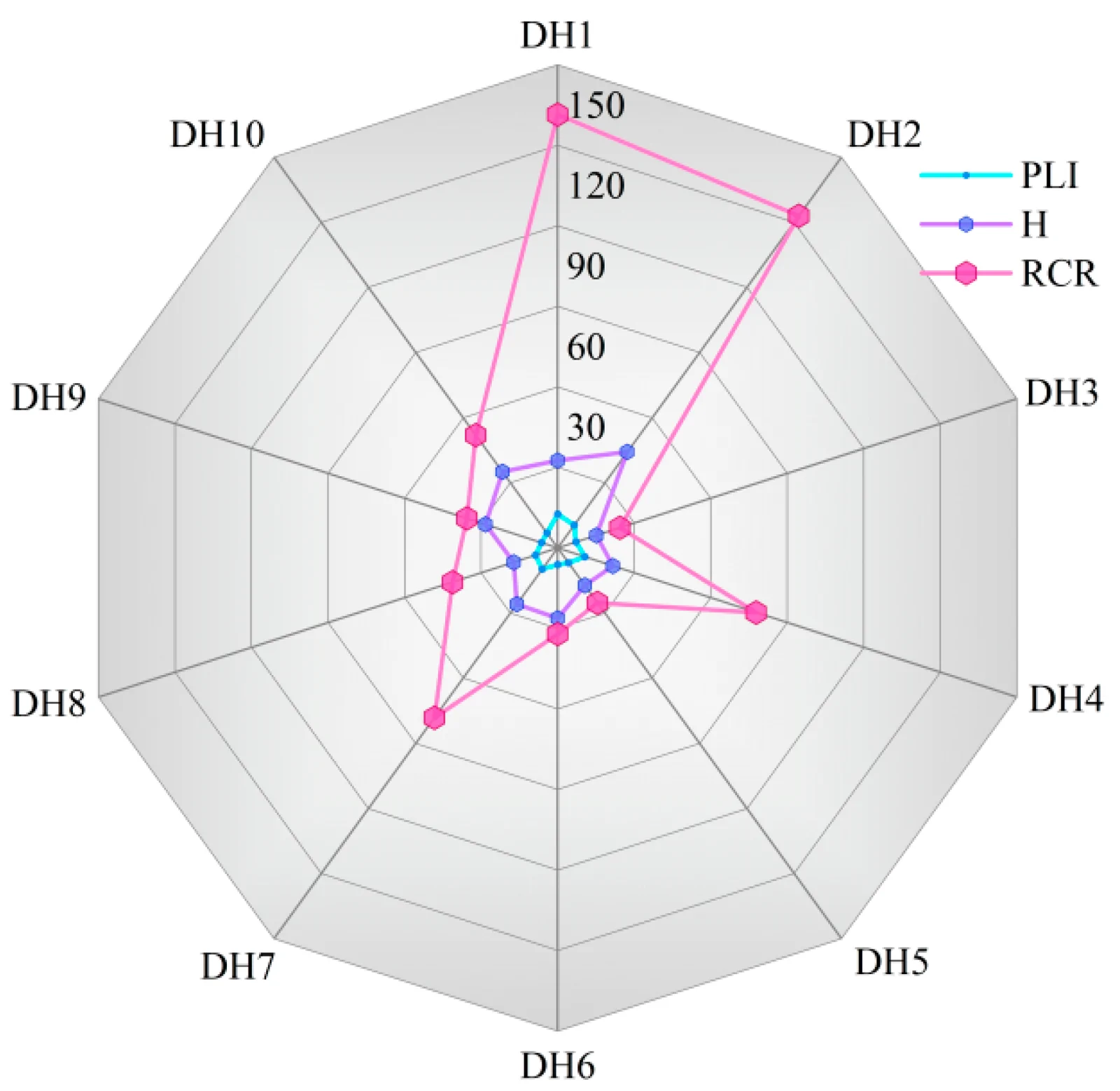
MPs risk assessment.

The PLI values at the Daihai site ranged from 6 to 13 (Figure 8), with three sites classified as level II risk and the remaining sites classified as level I risk. The average PLI value of sediments in the study area was 8, corresponding to a low pollution level. The RCR calculations showed that the RCR values of sediment MPs ranged from 24 to 162, with an average value of 68, indicating the presence of two high ecological risk sites. The highest PLI and RCR values in the study area were observed at the coastal sites DH1 and DH2, which were attributed to their proximity to residential areas, farmland, tourist areas, and power plants. These results suggested that human activities and industrial production were important sources of MPs input into the lake system.

The results showed that the PLI and RCR models exhibited limited consistency, which was mainly attributed to the influence of the H value [74]. The adsorption of HMs and other persistent organic pollutants onto MPs surfaces increased their potential toxicity, which was not fully considered in these models, resulting in differences in the ecological risk assessment of MPs in sediments. However, the calculated values can still provide some reference for the ecological risk assessment of MPs in the study area. Although the degree of MPs pollution in the study area was relatively low, whether these MPs will infiltrate into groundwater and participate in a larger hydrological cycle is a worthwhile research question, and the ecological effects they bring are meaningful for long-term monitoring and evaluation.

#### 3.4.3. Risk Assessment of MPs-HMs Combined Pollution

**Figure 9:**
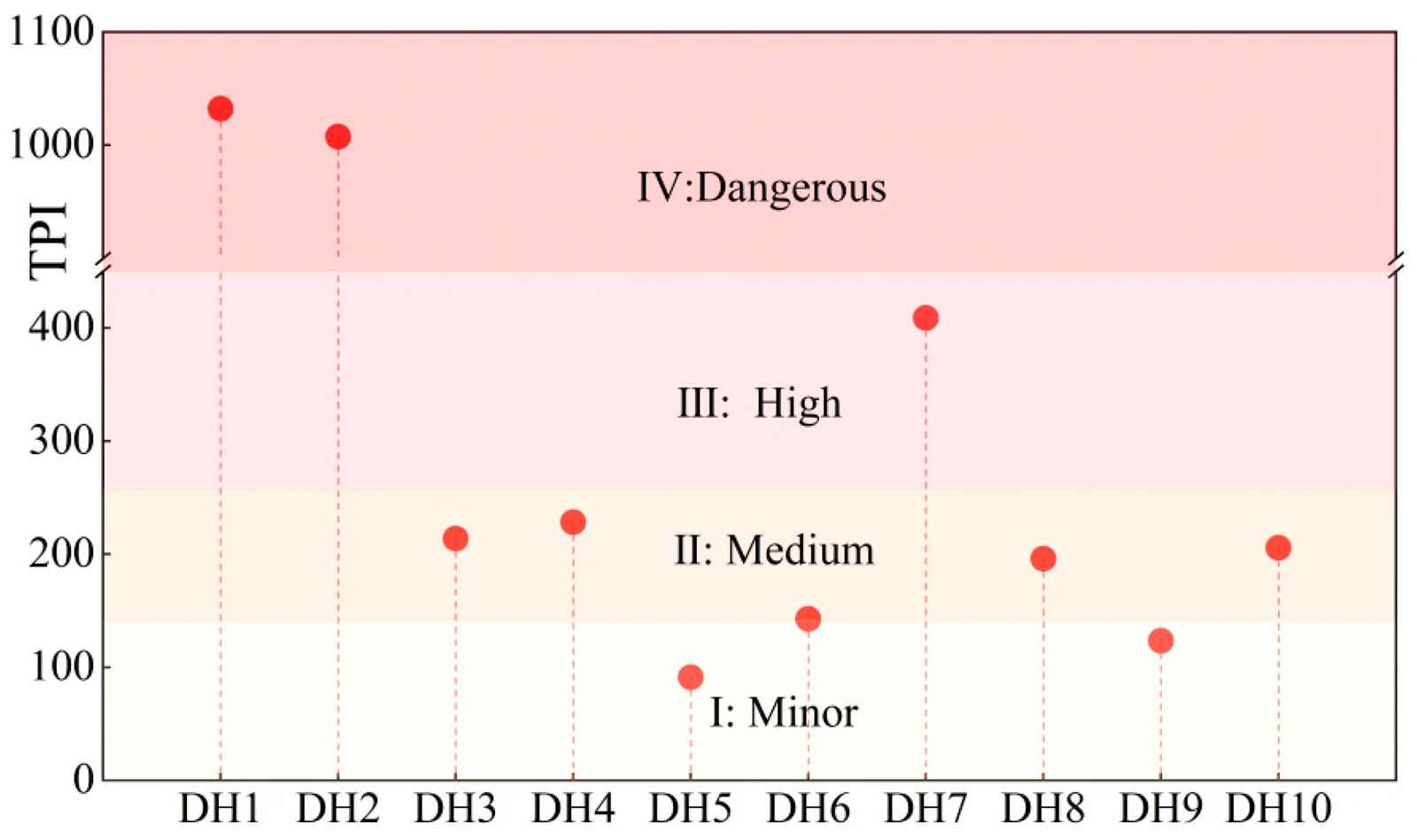
Risk level of comprehensive assessment of MPs-HMs.

Although the independent ecological risks of MPs and HMs have received widespread attention [38], there is still a lack of systematic assessment of their combined pollution behavior and joint ecological risks in lake environments. Regarding the sediments of Daihai Lake, this study is the first to construct an ecological risk assessment framework for MPs-HMs composite systems based on the comprehensive characterization of the morphological characteristics (shape, particle size, color) and polymer composition of MPs, and to quantify their combined pollution level (Figure 9). The TPI results showed that 70% of the sampling sites were classified as hazardous, 20% reached the extremely hazardous level, and one site exhibited a moderate risk level. The TPI values in the study area ranged from 124 to 1032, with an average value of 365, which was significantly higher than the risk warning threshold (TPI > 100). These results indicated that the lake system was under considerable stress from MPs-HMs composite pollution. The coexistence of MPs and HMs enhanced pollutant migration and bioavailability, thereby increasing the overall ecological risk and highlighting the necessity of collaborative management of composite pollution.

Sediments are important sinks for various pollutants in lake ecosystems, and MPs and HMs can be enriched in sediments through both sedimentation and adsorption processes [75]. As a terminal lake on the Inner Mongolian Plateau, Daihai Lake mainly relies on river inflow to maintain its water level. However, due to the evaporation exceeding the supply, the water level continues to decline. Lakes have no surface discharge outlets and strong hydrological sealing, which leads to the continuous concentration of HMs in the water and their adsorption by sediments. At the same time, MPs are continuously input through tributary runoff and enriched in the lake. In addition to their own toxicity, the interaction between MPs and coexisting HMs may change their migration behavior and bioavailability in the sediment environment, exacerbate pollutant loads, and continuously increase ecological risks [76], threatening the stability and health of lake ecosystems [66]. However, there is currently no standardized ecological risk assessment model for the combination of MPs and HMs, and there is still a lack of comprehensive research and systematic evaluation methods. Although this study used a two-dimensional evaluation framework, the mechanism of the interaction between MPs and HMs was not included in the evaluation system, which is also one of the directions worth studying in the future.

## 4. Conclusions

This study systematically investigated the occurrence characteristics, interactions, and ecological risks of MPs and HMs in sediments of Daihai Lake. The results demonstrated that MPs were mainly characterized by small-sized fragments, with a decreasing spatial trend from southwest to northeast, while Cd represented the primary contributor to HMs ecological risk. By integrating MPs characteristics and HMs toxicity, the composite risk assessment revealed that MPs-HMs co-pollution exhibited substantially higher ecological risks than individual pollutants. Significant associations between specific MPs characteristics (especially small-sized and fragmented particles) and HMs indicated their potential role as pollutant carriers. These findings highlight the importance of considering MPs-mediated interactions in sediment pollution risk assessments. Further studies combining advanced spectroscopic characterization, quantitative modeling, and microbial-mediated processes are needed to clarify the long-term evolution mechanisms of MPs-HMs coupling in aquatic ecosystems.

## Data Availability

The original contributions presented in this study are included in the article. Further inquiries can be directed to the corresponding author.

## Supplementary Materials

The following supporting information can be downloaded at https://www.mdpi.com/article/10.3390/toxics14080731/s1, Text S1. Experimental steps for HMs; Text S2. Analysis of the effectiveness of HMs; Table S1. Risk level standard for pollution load index; Table S2. RCR ecological risk level of MPs; Table S3. Application of plastic polymers, hazard index, and adjustment of hazard index; Table S4. MPs Risk Index (H) hazard Level; Table S5. RI risk assessment form; Table S6. MPs characteristic scoring; Table S7. MPERI Pollution degree criteria; Table S8. Research progress on sediments in different regions; Table S9. Coefficient of variation in HMs; Figure S1. MPs microscope photo; Figure S2. Horizontal spatial distribution of different particle sizes of MPs in sediments of Daihai Lake; Figure S3. Horizontal spatial distribution of different shape of MPs in sediments of Daihai Lake; Figure S4. Color composition of MPs; Figure S5. Changes in HMs concentration; Figure S6. Study on the forms of HMs; Figure S7. Pollutant clustering analysis; Figure S8. Concentration and effectiveness of HMs in sediment of Daihai Lake; Figure S9. Proportion of microplastic components at each point. References [77,78,79,80,81,82,83,84,85,86] cite in Supplementary Materials.

## Abbreviations

The following abbreviations are used in this manuscript:

MPs	microplastics
PP	polypropylene
PET	polyethylene terephthalate
PE	polyethylene
PVC	polyvinyl chloride
PS	polystyrene
FTIR	Fourier Transform Infrared Spectroscopy
PLI	Pollution Load Index
RCR	Risk Characterization Ratio
RI	Potential Ecological Risk Index
RSP	Comparison of Secondary and Primary Phases
RAC	Risk Assessment Coding Method
Igeo	Accumulative Index
MPERI	Multi Feature Potential Ecological Risk Index
TPI	Two-dimensional comprehensive index
QA	quality assurance
QC	quality control
RSD	relative standard deviation
CV	coefficient of variation
EPS	Extracellular polymers
ICP-MS	Inductively coupled plasma mass spectrometry
