# Supplementary material for: Coexistence of Microplastics and Heavy Metals in Lake Sediments: Interaction Mechanisms and Complex Ecological Risks
Source: Toxics. 2026 Aug 18;14(8):731. doi: 10.3390/toxics14080731 (PMC13517747; doi:10.3390/toxics14080731)

Supporting Materials

Coexistence of microplastics and heavy metals in lake sediments: interaction mechanisms and complex ecological risks

Jiahui Mi[b], Junping Lu[a,b,c,d], Zhuo Li[b], and Yongqin Jia[a,b,c,d*]*

[a] *State Key Laboratory of Water Engineering Ecology and Environment in Arid Area, Inner Mongolia Agricultural University, Hohhot 010018, China*

[b] *Water Conservancy and Civil Engineering College, Inner Mongolia Agricultural University, Hohhot 010018, China*

[c] *State Gauge and Research Station of Wetland Ecosystem, Wuliangsuhai Lake, Bayan Nur, Inner Mongolia 014404, China*

[d] *Autonomous Region Collaborative Innovation Center for Integrated Management of Water Resources and Water Environment in the Inner Mongolia Reaches of the Yellow River, Hohhot 010018, China*

[*]Corresponding authors.

Email address: lujunping2008@imau.edu.cn (J. Lu)
Email address: jiayongqin0471@163.com

Number of Pages (including the cover): 15

Number of Texts: 2

Number of Figures: 8

Number of Table: 8

List of content:

Text S1 Experimental steps for HMs

Text S2 Analysis of the Bioavailability of HMs

Table S1. Risk level standard for pollution load index

Table S2. RCR ecological risk level of MPs

Table S3. Application of plastic polymers, hazard index, and adjustment of hazard index

Table S4. MPs Risk Index (H) hazard Level

Table S5. RI risk assessment form

Table S6. MPs characteristic scoring

Table S7. MPERI Pollution degree criteria

Table S8. Research progress on sediments in different regions

Table S9. Coefficient of variation of HMs

Fig. S1. MPs microscope photo

Fig. S2. Horizontal spatial distribution of different particle sizes of MPs in sediments of Daihai Lake

Fig. S3. Horizontal spatial distribution of different shape of MPs in sediments of Daihai Lake

Fig. S4. Color composition of MPs

Fig. S5. Changes in HMs concentration

Fig. S6. Study on the forms of HMs

Fig. S7. Pollutant clustering analysis

Fig. S8. Concentration and bioavailability of HMs in sediment of Daihai Lake

Fig. S9. Proportion of microplastic components at each point

Text S1 Experimental steps for HMs

Weigh 1 g of air dried and sieved sample and place it in a digestion tank. Rinse it with deionized water twice. Add 6 ml of nitric acid, 3 ml of hydrochloric acid, and 2 ml of hydrofluoric acid in an acid resistant fume hood. Mix the sample and digestion solution thoroughly. After the reaction is complete, tighten the digestion tank and place it in a microwave digestion oven. Set the heating program: raise the temperature from room temperature to 120°C for 7 min, hold it at 120°C for 3 min, then heat it up for 5 min, raise it from 120°C to 160°C, hold it at 160°C for 3 min, and finally heat it up for 5 min, raise it to 190°C and hold it for 25 min, after cooling to room temperature, remove the digestion tank and place it in an acid resistant fume hood. Slowly release the pressure and release the gas. Open the digestion tank and transfer the solution to a polytetrafluoroethylene crucible. Wash the inside and lid of the digestion tank with a small amount of 5% nitric acid solution and pour them into the crucible together. Set the temperature of the temperature controlled electric heating plate to 240°C and perform acid quenching in a slightly boiling state. When the liquid is almost dry and viscous, remove and cool it. Use a dropper to drip 5% nitric acid and rinse the inner wall of the crucible. Use the remaining temperature to dissolve the residue attached to the crucible wall and transfer it to a 25 ml volumetric flask. Repeat the washing with 5% nitric acid and transfer it to the volumetric flask. Then, dilute to the mark with 5% nitric acid, mix well, and let it stand for 1 h to obtain the supernatant for testing. Wet settled samples are filtered through a 0.45 μm membrane to remove impurities for testing.

ICP-MS is used for the determination of total HMs content. Compared with other traditional techniques, ICP-MS technology has the lowest detection limit and the highest linear range, fast analysis speed, high precision, and can simultaneously analyze more than 70 elements. It is widely

used in many fields such as environment, semiconductor, medicine, and physiological analysis. The preprocessed samples will be detected using an inductively coupled plasma mass spectrometer (NexION 1000), and qualitative analysis will be performed based on the mass spectra or characteristic ions of the elements, followed by quantitative analysis using internal standard method. To reduce errors, this experiment used the national standard soil sample GSS-8 (GBW-07408), blank sample, and parallel sample for quality control. The recovery rate error of HMs in the standard sample was less than 5%.

The different chemical forms of HMs are separated using the BCR stepwise extraction method provided by the European Community Bureau of Standards and Materials. The steps for extracting the different forms of HMs are as follows:

(1) Acid extractable form (F1) state: Take 0.25 g of the sample and place it in a 100 ml polyethylene centrifuge tube. Add 10 ml of CH_3COOH solution with a concentration of 0.11 mol·L^{-1}. Tighten the tube plug and place it in a constant temperature oscillation box. Set the temperature to 25°C and the speed to 30 rpm for 16 h of constant temperature oscillation. Then centrifuge at 4000 rpm for 20 min in a high-speed centrifuge. Take the supernatant and filter it through a 0.45 μm filter membrane for testing. Wash the residue with 10 ml of deionized water, centrifuge for 20 min, and discard the washing solution.

(2) Reducible state (F2) state: Add 10 ml of NH_2OH·HCI with a concentration of 0.1 mol·L^{-1} (adjusted to pH=2 with dilute HNO_3 solution) to the residue from the previous step, tighten the tube plug and place it in a constant temperature oscillation box. Set the temperature to 25°C and the speed to 30 rpm for 16 h of constant temperature oscillation. Then centrifuge at 4000 rpm for 20 min in a high - speed centrifuge and wait for testing. The other steps are the same as above.

(3) Oxidizable state (F3) state: Add 2.5 ml of 8.8 mol·L^{-1} H_2O_2 to the residue from the previous

step, loosely cover the centrifuge tube to prevent significant loss of hydrogen peroxide, and oscillate intermittently for 1 h under 25℃ water bath heating conditions. Remove the lid and heat it in an 85℃ water bath for 1 h, then add 2.5 ml of 8.8 mol·L^{-1} H_2O_2 and continue to heat it in the water bath until the solution is almost dry. Add 12.5 ml of 1 mol·L^{-1} NH_4OAc (adjusted to pH 2 with dilute HNO_3 solution) and place it in a constant temperature oscillation box. Set the temperature to 25℃ and the speed to 30 rpm for continuous oscillation for 18 h, then centrifuge at 4000 rpm for 20 min. Take the supernatant and filter it through a 0.45 μm membrane for testing. The other steps are the same as before.

(4) Residual state (F4) state: Dissolve the residue from the previous step according to the "HJ 832-2017 Microwave Digestion Method for Total Metal Content in Soil and Sediments". After digestion, dilute the sample with 5% HNO_3 to 25 ml, mix well, and let it stand for 1 h. Take the supernatant for testing.

Text S2 Analysis of the bioavailability of HMs

The sum of the HMs content obtained in the first three steps of this experiment represents the effective portion of HMs, this three components are considered as bioavailable states (AHMS) in sediments, AHMS of HMs in sediments is relatively easy to release in the environment, has high migration ability, is easily absorbed by passive plants, and has the highest degree of biological harm and toxicity [77]. Among them, F1 form is the most sensitive in the environment, with strong mobility and easy release, which can cause harm to the environment and organisms [78]; When the redox conditions of sediment change, the F2 and F3 form will be released into the environment, which will be passively absorbed by plants and cause harm to the environment. Fig.S8 summarizes the AHMS of eight HMs elements, among which Cd is mainly in the non residual state

(F1+F2+F3), and its bioavailability relative to their respective total amounts is the highest among the 8 HMs AHMS, at 77.06%. However, the non residual state is easily released under acidic conditions and can be toxic to the environment and benthic organisms [79]. Therefore, Cd has strong toxicity to the environment and organisms. The utilization rate of Mn element has decreased significantly to 24.30%, indicating that Mn element is less affected by human activities. The utilization rates of the remaining 6 elements are in the range of below 2.00%, indicating that the residual content of Cr, Cu, Zn, Ni, and As is very high, mainly distributed in the silicate lattice, with relatively stable properties that are not easily absorbed by organisms, thus reducing the harm to the environment.

Table S1. Risk level standard for pollution load index

Risk level	I	II	III	IV
MPs pollution meets the index	<10	10-20	20-30	>30

Table S2. RCR ecological risk level of MPs

Project	0<RCR≤1	1<RCR≤10	10RCR≤100	RCR>100
Ecological hazard level	I	II	III	V

Table S3. Application of plastic polymers, hazard index, and adjustment of hazard index

MPs type	Polymer abbreviation	Hazard index(S_i)	Adjust hazard index(Z_i)
Polyvinyl chloride	PVC	10001	666.730
Polystyrene	PS	30	2.000
Polyethylene	PE	11	0.733
Polyethylene terephthalate	PET	4	0.267
Polypropylene	PP	1	0.067

Table S4. MPs Risk Index (H) hazard Level

Project	0<H≤1	1<H≤10	10<H≤100	100<H≤1000	H>1000
Ecological hazard level	I	II	III	IV	V

Table S5. RI risk assessment form

Project	HMs							
	Cr	Ni	Pb	Cu	Zn	Mn	As	Cd
Toxicity coefficient	2	5	5	5	1	1	10	30
	Ecological hazard index							
Risk level	$E_r^i \leq 40$		$40 < E_r^i \leq 80$	$80 < E_r^i \leq 160$		$160 < E_r^i \leq 320$		$E_r^i > 320$

	Mild	Moderate	Medium height	Height	Extremely high
	Comprehensive potential ecological risk index				
Risk level	RI ≤ 150	150 < RI ≤ 300	300 < RI ≤ 600	RI > 600	
	Mild	Medium height	Height	Extremely high	

Table S6. MPs characteristic scoring

Characteristics of MPs	Shape			Color					
	Fibrous	Fragment	Film	Yellow	Red	Green	Black	White	Blue
Rating	1	0.83	0.88	1	0.65	0.61	0.57	0.57	0.35

Table S7. MPERI Pollution degree criteria

Grade	Minor	Medium	High	Dangerous	Extremely dangerous
Rating	MPERI < 10	10 ≤ MPERI < 100	100 ≤ MPERI < 500	500 ≤ MPERI < 1000	MPERI ≥ 1000
Rating	TPI < 120	120 ⩽ TPI < 255	255 ⩽ TPI < 563	563 ⩽ TPI < 1151	TPI ⩾ 1151

Table S8. Research progress on sediments in different regions

MPs particles were found in all sediment sampling points in Daihai, with a MPs abundance ranging from 6.60-26.80 $n\cdot g^{-1}$, indicating that sediments are one of the storage sites for MPs. Comparing the research on MPs in sediment at home and abroad with this study, it was found that the abundance of MPs in this study is relatively high, and their sizes are mainly concentrated in the range of less than 0.25 mm. In contrast, MPs in sediment from foreign lakes are mainly concentrated in the range of around 1.00 mm, which is different from other studies where MPs mainly exist in fibrous form. The main shape of MPs in sediment in this study is fragmented, followed by fibrous form.

Region	Main size range	Main shape	Abundance	References
Bangladesh	Less than 0.50mm	Fibrous	12.50-55.00 n·kg^{-1}	[80]
Jiangsu	10.00 -100.00 cm	Fibrous	0.20-12.50 n·g^{-1}	[81]
Tibet	1.00 - 5.00 mm	--	8.00-563.00 n·m²	[82]
United States	--	Fragmented	3209.00 n	[83]
Canada	0.78 - 3.00 mm	--	483.00-1108.00 n·m²	[84]
India	--	Fibrous	96.00-496.00 n·m²	[85]
Inner Mongolia	Less than 0.25mm	Fragmented	6.60-26.80 n·g^{-1}	This study

Table S9. Coefficient of variation of HMs

Among them: CV<10%: low variability (possibly controlled by natural background); 10% ≤ CV<30%: moderate variability; CV ≥ 30%: high variability (usually strongly influenced by human interference).

HMs	Cd	Pb	As	Cr	Ni	Cu	Mn	Zn
CV(%)	57.73	8.36	19.84	22.95	17.66	8.06	10.34	10.34

Fig. S1. MPs microscopy image

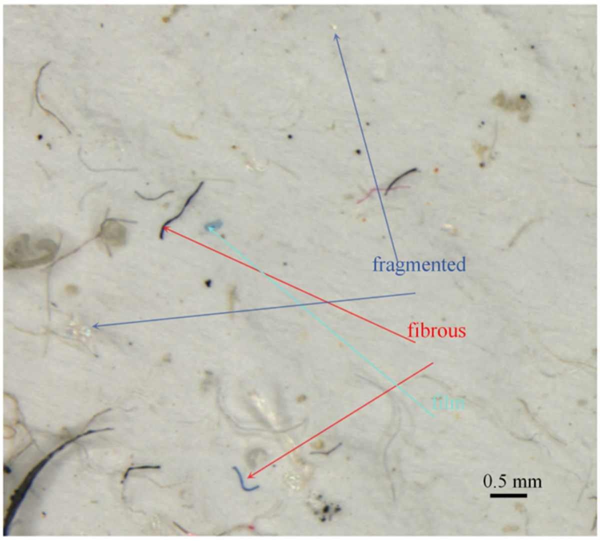

Fig. S2. Horizontal spatial distribution of different particle sizes of MPs in sediments of Daihai Lake

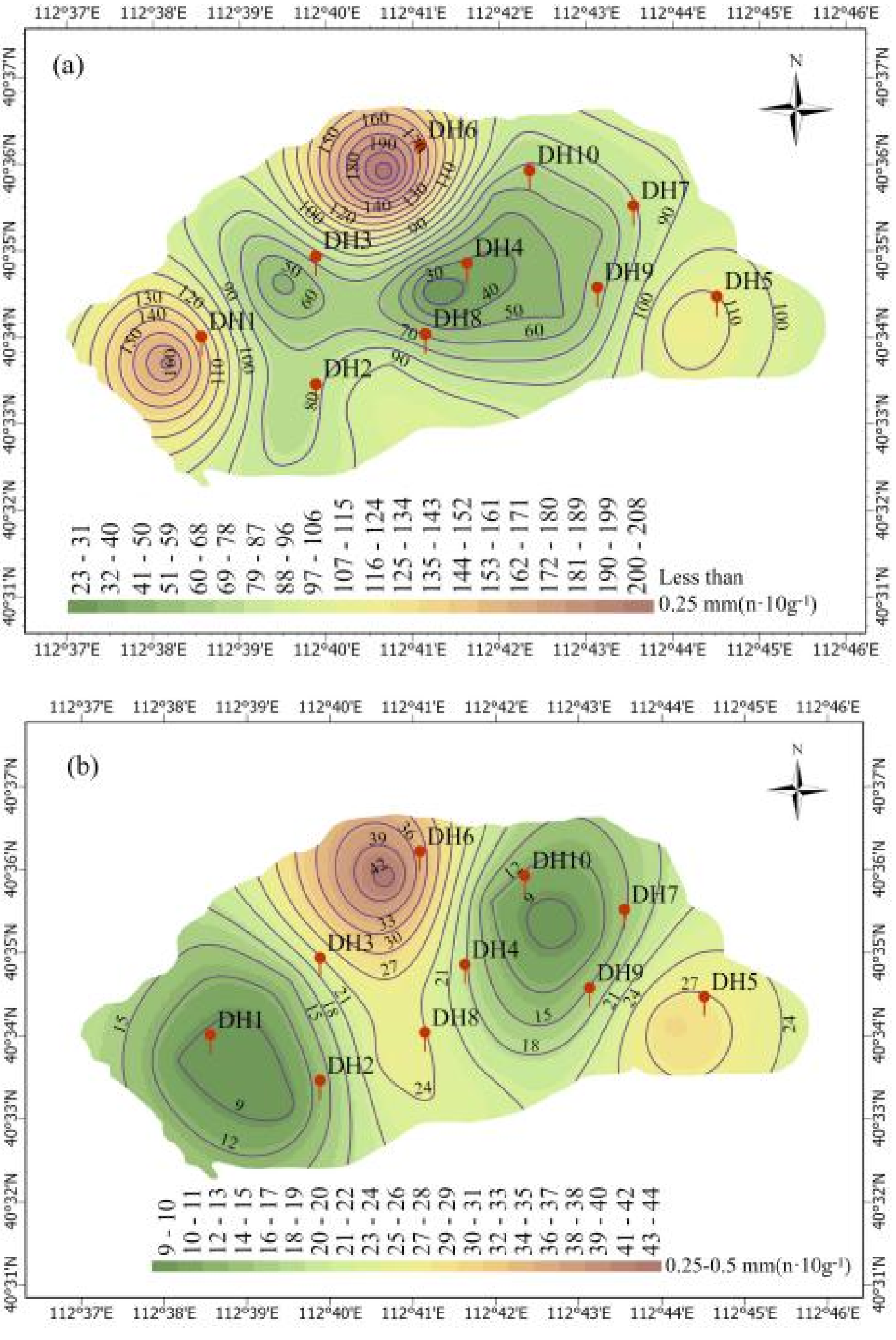

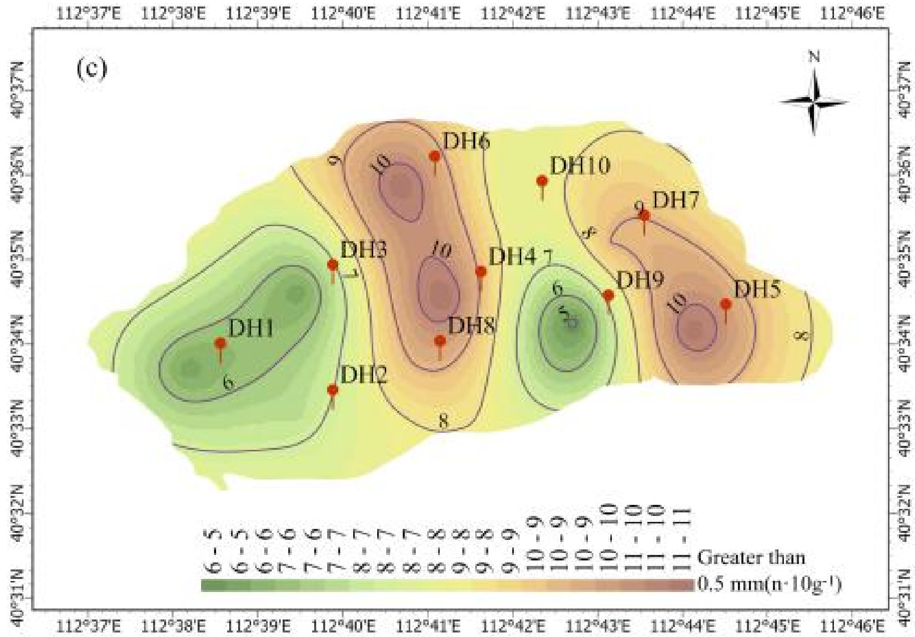

(a) less than 0.25 mm, (b) 0.25 - 0.50 mm, (c) greater than 0.50 mm.

Fig. S3. Horizontal spatial distribution of different shape of MPs in sediments of Daihai Lake

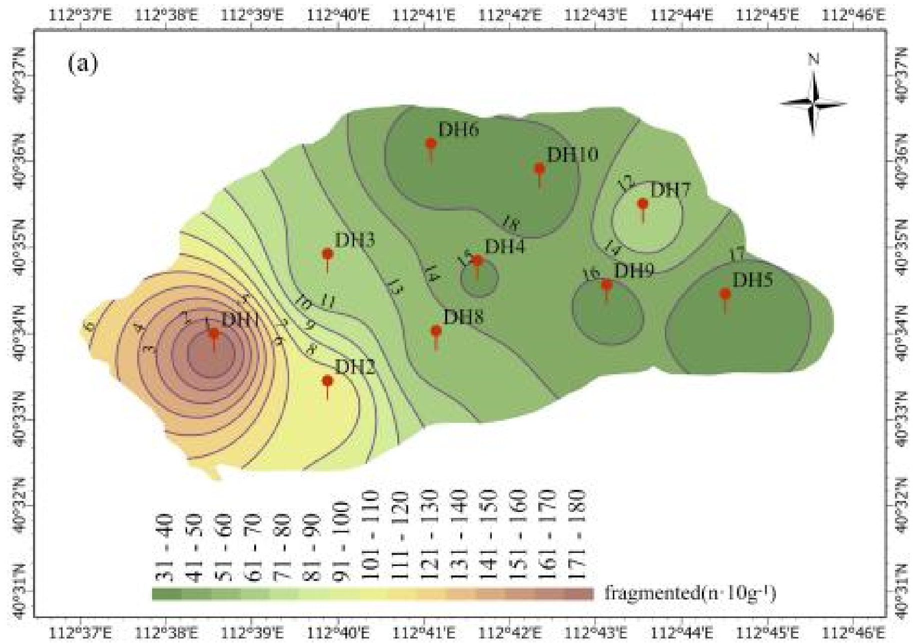

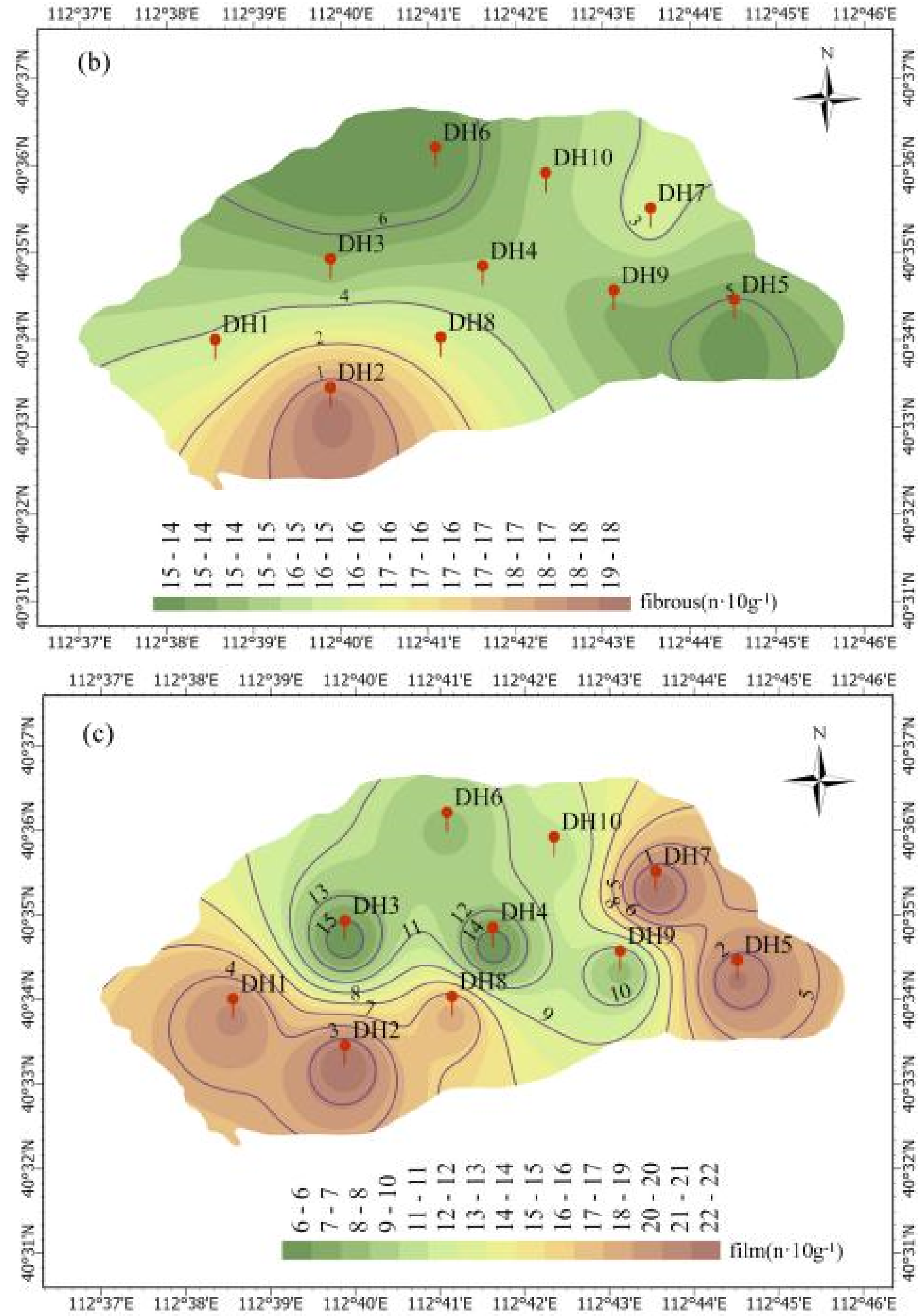

(a) fragmented, (b) fibrous, (c) film.

Fig. S4. Color composition of MPs

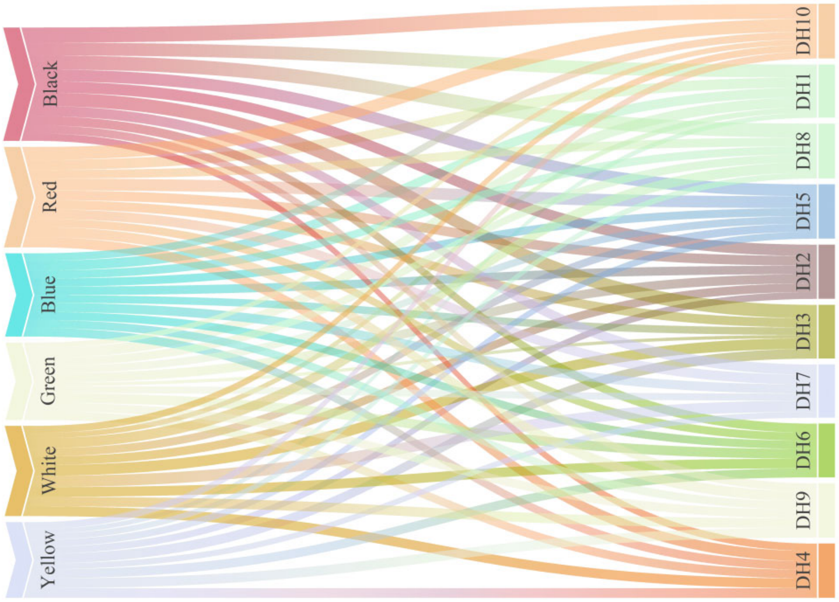

Fig. S5. Changes in HMs concentration

There are certain spatial differences in the concentration of HMs, with DH3 having the highest concentration due to the impact of non-point source pollution such as tourism and surrounding farmland. DH1, DH2, and DH8 have severe point source pollution and relatively high concentrations of HMs due to their proximity to power plants on the shore. DH4 and DH9, located near the center of the lake, have the lowest concentrations due to their distance from industrial areas on land and minimal human impact. The overall trend of HMs concentration in the sediment of Daihai Lake is decreasing from the lake edge to the lake center and from west to east

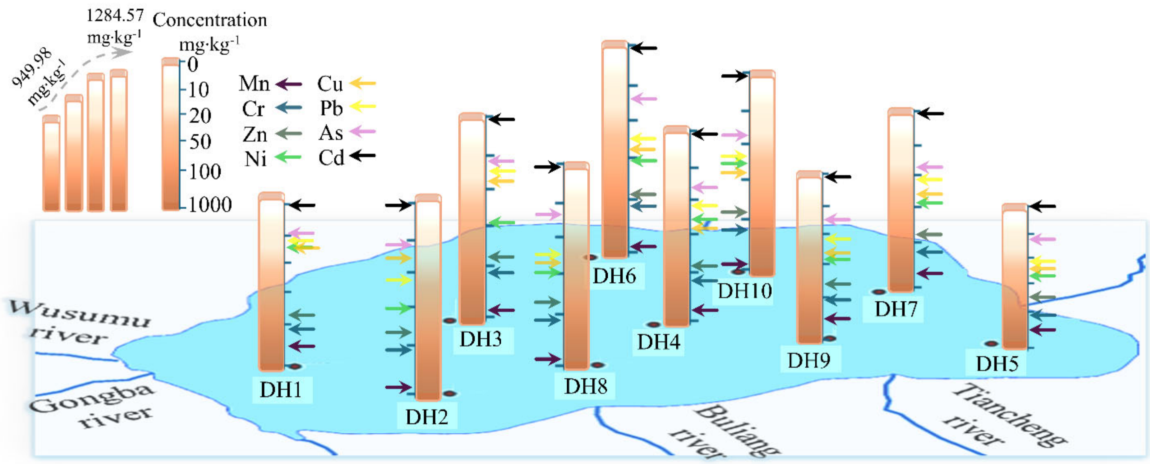

Fig. S6. Study on the forms of HMs

The different occurrence forms of HMs in sediments can not only reveal the migration ability of HMs, but also reflect the toxic hazards of HMs to the ecological environment [86].

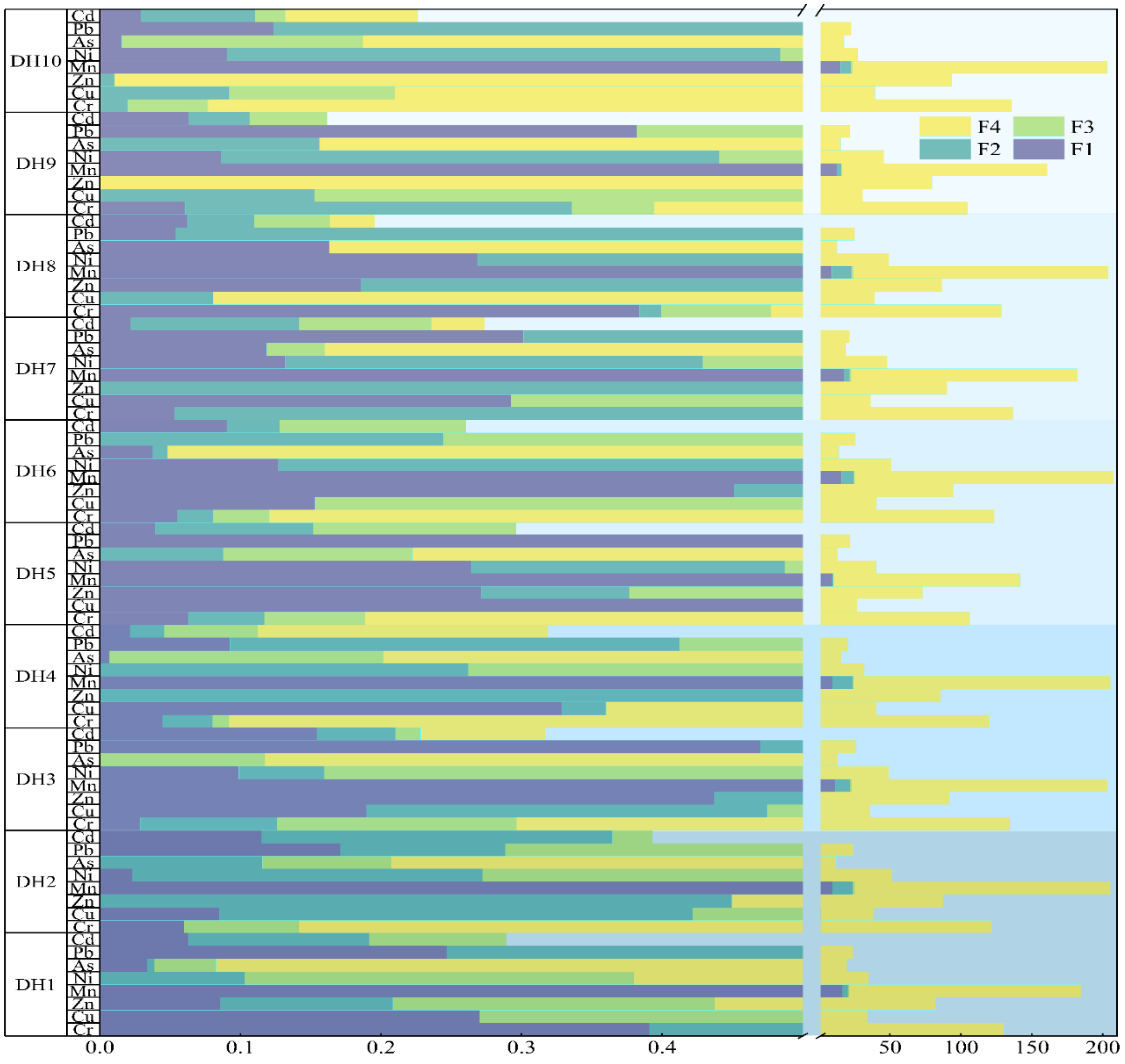

Fig. S7. Pollutant clustering analysis

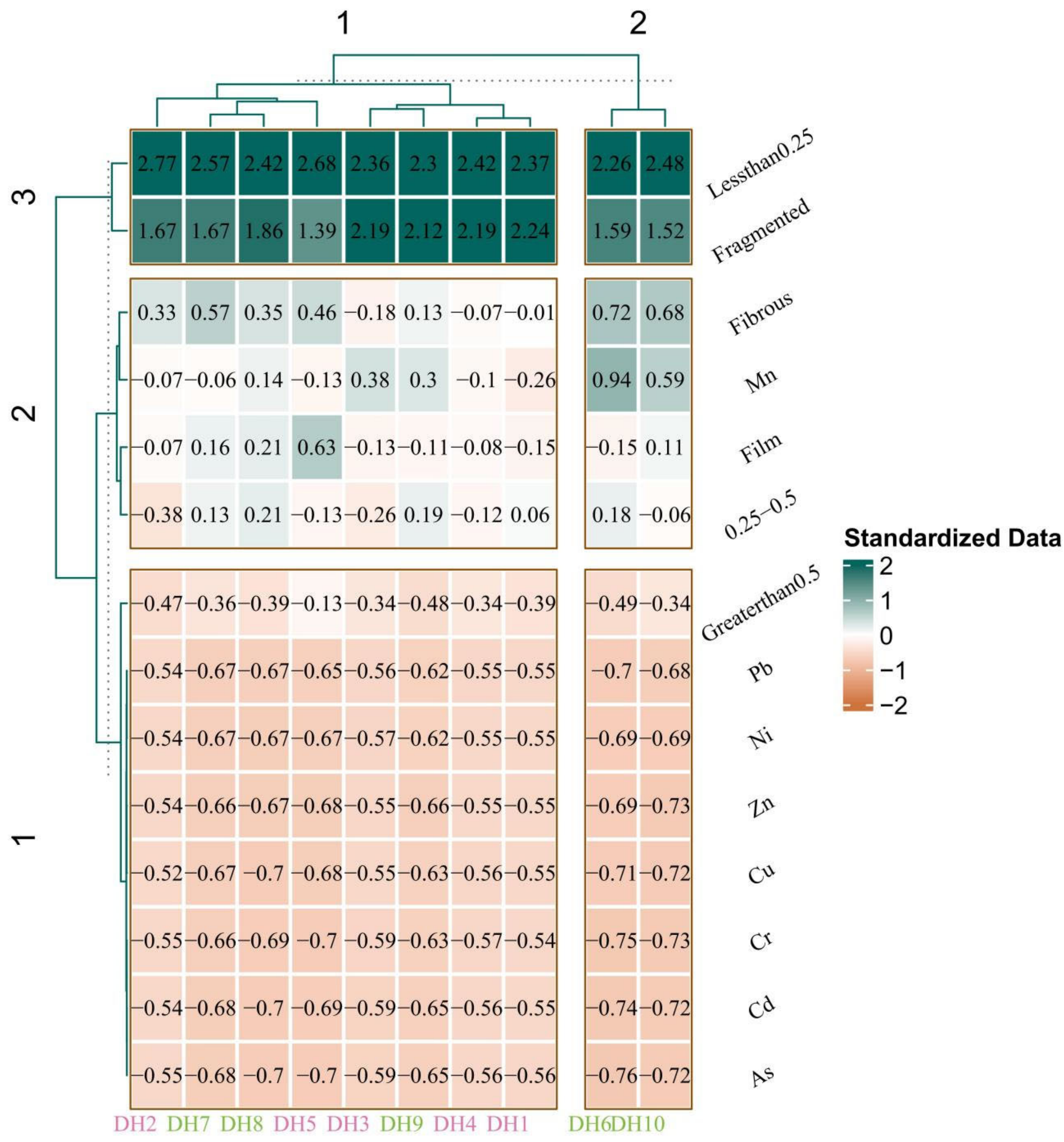

Fig. S8. Concentration and Bioavailability of HMs in sediment of Daihai Lake

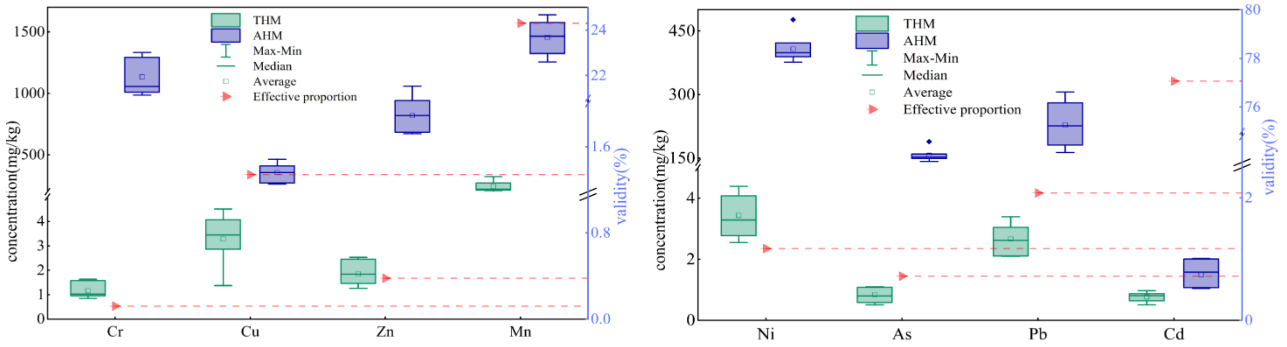

Fig. S9. Proportion of microplastic components at each point

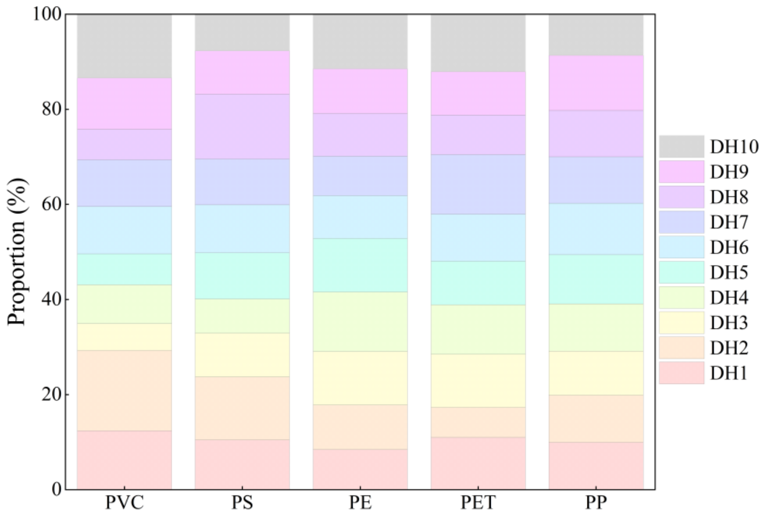

Supplement: Supplementary file 1 [file toxics-14-00731-s001.zip › toxics-4448176-supplementary.pdf]
